# Comprehensive Analysis of LANA Interacting Proteins Essential for Viral Genome Tethering and Persistence

**DOI:** 10.1371/journal.pone.0074662

**Published:** 2013-09-11

**Authors:** Subhash C. Verma, Qiliang Cai, Edward Kreider, Jie Lu, Erle S. Robertson

**Affiliations:** 1 Department of Microbiology and Immunology, School of Medicine, University of Nevada, Reno, Nevada, United States of America; 2 MOE& MOH Key Laboratory of Medical Molecular Virology, School of Basic Medicine of Fudan University, Shanghai, China; 3 Department of Microbiology and Tumor Virology Program of the Abramson Comprehensive Cancer Center, Perelman School of Medicine at the University of Pennsylvania, Philadelphia, Pennsylvania, United States of America; University of Nebraska - Lincoln, United States of America

## Abstract

Kaposi’s sarcoma associated herpesvirus is tightly linked to multiple human malignancies including Kaposi’s sarcoma (KS), Primary Effusion Lymphoma (PEL) and Multicentric Castleman’s Disease (MCD). KSHV like other herpesviruses establishes life-long latency in the infected host by persisting as chromatin and tethering to host chromatin through the virally encoded protein Latency Associated Nuclear Antigen (LANA). LANA, a multifunctional protein, is capable of binding to a large number of cellular proteins responsible for transcriptional regulation of various cellular and viral pathways involved in blocking cell death and promoting cell proliferation. This leads to enhanced cell division and replication of the viral genome, which segregates faithfully in the dividing tumor cells. The mechanism of genome segregation is well known and the binding of LANA to nucleosomal proteins, throughout the cell cycle, suggests that these interactions play an important role in efficient segregation. Various biochemical methods have identified a large number of LANA binding proteins, including histone H2A/H2B, histone H1, MeCP2, DEK, CENP-F, NuMA, Bub1, HP-1, and Brd4. These nucleosomal proteins may have various functions in tethering of the viral genome during specific phases of the viral life cycle. Therefore, we performed a comprehensive analysis of their interaction with LANA using a number of different assays. We show that LANA binds to core nucleosomal histones and also associates with other host chromatin proteins including histone H1 and high mobility group proteins (HMGs). We used various biochemical assays including co-immunoprecipitation and in-vivo localization by split GFP and fluorescence resonance energy transfer (FRET) to demonstrate their association.

## Introduction

Kaposi’s sarcoma associated herpesvirus also known as human herpesvirus 8 (HHV8), is a member of the gammaherpesvirus family and is the causative agent of multiple lymphoproliferative diseases including Kaposi’s sarcoma (KS), Body Cavity Based Lymphomas (BCBLs) and Multicentric Castleman’s Disease (MCDs). KSHV, like other herpesviruses establishes lifelong latent infection with its genome persisting as extra-chromosomal episomes [1]–[3]. During latency only a limited number of viral genes are expressed, which helps in viral genome replication and persistence without being recognized by the host immune system [4]–[6]. Latency Associated Nuclear Antigen (LANA) is one of the major proteins expressed in all latently infected cells [2], [7], [8]. LANA is considered to be an oncogenic protein because it can modulate various cellular pathways involved in the induction of tumorigenesis [9]–[12]. LANA achieves this by degrading tumor suppressors, p53, pRb and von Hippel Lindau (VHL) by ubiquitinating them through the recruitment of ubiquitin ligases [10], [13]–[15]. Besides degrading tumor suppressors, LANA upregulates the expression of proteins important for cell immortalization including upregulation of human telomerase, hTERT [9], [16]. LANA is also important for physically tethering the viral genome to the host chromosome and segregation of viral episomes to daughter cells to maintain an almost constant copy number of viral genomes in dividing tumor cells [2], [3], [17].

KSHV genome deleted for the LANA gene was unable to establish latent infection due to the lack of persistence in the target cells [17]. Additionally, depletion of LANA by shRNA in PEL cells showed depletion of viral genome copies compared to the cells treated with control [18]. These studies clearly indicated that LANA is important for the persistence of viral DNA. To achieve tethering and efficient segregation into the daughter cells LANA binds to various cellular proteins [2], [19]–[26]. From the time LANA was detected as a nuclear protein, studies were carried out to determine its role in viral genome persistence [2], [3], [27]. Pioneering studies showed that LANA localizes to the nucleus of latently infected cells as punctate speckles and co-localizes with viral genomes on the host chromosomes [2], [3]. Further, LANA was shown to bind to linker histone, H1 and this interaction was proposed to be required for tethering onto the host chromosome [2]. Immediately after that the chromosome-targeting region of LANA was mapped to amino acids 1–32, and the deletion of this region was shown to inhibit interaction of LANA with mitotic chromosome [27]. Further studies were carried out to determine whether a chimeric LANA with histone H1 could be targeted to host chromosome and persist over multiple cell divisions [28]. The results of the study showed that LANA deleted for 1–22 aa were unable to target to chromatin and replicate terminal repeat (TR) containing plasmids, whereas Δ1–22 aa LANA fused with histone H1 bound to chromosomes as well as supported replication [28]. The association of LANA with chromatin-associated proteins RING3, which colocalizes to heterochromatin, and heterochromatin proteins 1 (HP1) was also detected by various biochemical assays [23], [29], [30]. Additional studies aimed at identifying the LANA interacting proteins determined binding of methyl CpG binding protein MeCP2 and 43 kDa protein DEK by GST binding and yeast 2-hybrid assays [19].

The landscape of LANA association with chromatin changed after interesting work from Kaye’s group which determined that chromosome binding domain (CBD) fused to green fluorescent proteins (GFP) uniformly stains the metaphase chromosome suggesting an abundance of LANA targets on the chromosomes [20]. Further biochemical and crystallographic experiments identified that CBD peptides of LANA docks onto the surface of nucleosome octamer by directly binding to core histones H2A/H2B subunits [20]. More recent studies identified double bromodomain binding protein, BRD4, BRD3 and Nuclear Mitotic Apparatus (NuMA) proteins as LANA interacting proteins [21], [25], [26], [31]. These proteins are important for tethering and efficient segregation of the viral genome. Indeed NuMA, which is essential for cellular genome segregation by interacting with microtubule dynein/dynactin during mitosis, colocalized with LANA during specific cell cycle phases [21]. Depletion of NuMA by siRNA and the use of dominant-negatives to block NuMA function showed reduction in latently maintained KSHV epiosomal copies [21]. We also showed that centromeric protein F (CENP-F), a component of the multiprotein complex, which assembles on centromeric DNA to link the chromosome to microtubules during mitosis, interacts with LANA [32]. We showed that LANA associates with CENP-F along with the kinetochore protein Bub1 and depletion of Bub1 by shRNA dramatically reduces latently persisting KSHV genome copies [32]. Therefore, these studies strongly suggested that KSHV episomes segregate in a similar fashion to the host genome at the time of host genome segregation. However, the exact mechanism of genome tethering and segregation may be more complex as the expression of LANA was not enough to retain the TR containing plasmids without drug selection [33]. This suggests contribution by more than one of the above listed proteins as well as viral factors in tethering and segregation of the viral genome.

In this report, we sought to comprehensively determine the relative binding of core and linker histones along with identification of additional chromatin binding proteins to LANA. We used co-immunoprecipitation assays on the lysates from latently infected KSHV PEL cells treated with micrococcal nuclease (to generate mono-nucleosomes by degrading inter-nucleosomal DNA) and lysates treated with DNase I (to disrupt secondary association due to DNA) from over-expressed and stably selected cells. We generated alanine substitution mutants in CBD of LANA to determine the specific association with histone H1 and H2B expressed in eukaryotic cells as well as translated *in-vitro*. We used *in-vivo* protein-protein interaction assays including split GFP complementation and fluorescence resonance energy transfer (FRET) to demonstrate LANA’s binding to the components of cellular chromatin. In agreement with the results of prior studies [20], we found that LANA associates with histone H2B both *in-vitro* as well as *in-vivo* along with the linker histone, H1. We also identified high mobility group N1 (HMG-N1) protein as a LANA interacting partner, which may cooperate in tethering to the host chromatin.

## Materials and Methods

### Plasmids, Cell lines and Antibodies

GFP-NLS-myc was constructed by inserting a duplex oligo (GATCC**aagaggcccaggagtcccagtagt**GGATCCgaacaaaagctgatttctgaagaagactt*g* gaacaaaagctgatttctgaagaagacttgT) containing Epstein-Barr Virus Nuclear Antigen 1 nuclear localization signals (bold and underlined) and two consecutive myc tags (one tag within the box second underlined) into a pEGFP-C1 at BamHI and XbaI site of MCS. GFP-LANA1–32 aa-myc was generated by inserting PCR amplified product of LANA 1–32 aa in-frame with GFP and myc tags at EcoRI and BamHI sites of GFP-NLS-myc. Digestion of GFP-NLS-myc with BamHI eliminated EBNA1 NLS, which was flanked by BamHI sites. pA3M-LANA (myc tagged), pA3F-LANA (flag tagged) and their deletion mutants containing myc-tagged LANA amino-terminal domain (1 to 340 aa), carboxy-terminal domain (940 to 1162 aa) were described previously [34]–[36]. Alanine substitution mutants, 5-GMR-7, 8-LRS-10, 11-GRS-13, 14-TG-15 and 5–15 aa to alanines in LANA 1–32, 1–340 and LANA full length were generated by site directed mutagenesis as described previously [20]. The GFP-LANA deletion constructs carrying myc tagged LANA 1–340 aa and 940 to 1162 aa were constructed by PCR amplification using LANA as template and inserting the PCR product in-frame with GFP and myc in GFP-NLS-myc vector. Histone H1 and H2B IMAGE clones were purchased from Invitrogen (H1-IMAGE:3538400 and H2B-IMAGE:5190019). Histone H1 sequence was identified as from Homo sapiens H1 histone family, member X. Specific primers were used for the amplification of histone H1 and H2B, using DNA from above IMAGE clones as template, to insert into pA3M (Myc-tagged), pA3F (Flag-tagged) and pCDNA3.1HA (HA-tagged) vectors. Yellow Fluorescent Protein tagged LANA (YFP-LANA-flag) was constructed by inserting LANA-flag from pA3F-LANA into pEYFP-C1 (Clontech) in frame with YFP. Similarly, the Cyan Fluorescent Protein tagged histones (CFP-H1-myc and CFP-H2B-myc) were generated by PCR amplifying and cloning histone H1-myc and H2B in-frame with CFP of pECFP-C1 (Clontech). Lentiviral construct pLVX-YFP-LANA-Flag was constructed by PCR amplification from LANA as template and inserting into pLVX-AcYFP-C1 (Clontech). Lentiviral vector expressing histone H1 and H2B in frame with GFP-myc were generated by PCR amplification using the above template and cloning the amplicon into pLVX-AcGFP-C1 vector (Clontech). Variants of histone H1 including H1.c (cat# RC201249), H1.e (cat# RC215208) and H1.b (cat# RC224189) were purchased from Origene (OriGene Technologies, Inc.). Flag tagged constructs of these histone H1 variants were generated by PCR amplification and cloning in frame with the flag tag into pA3F. GST fusion proteins of LANA 1–32 and its alanine mutants were generated by PCR amplification using above-mentioned respective templates of GFP-1-32myc clones into pGEX-2T vector (GE). Similarly, histone H1 and H2 were cloned into pGEX-2T in frame with GST by PCR amplifying using pA3M-H1 and pA3M-H2B templates. Sequences of each clone including the alanine substitution mutants were verified by sequence analysis. MeCP2 was a generous gift from Dr. Diane Hayward (Johns Hopkins), DEK was kindly provided by Dr. Gerald Grosveld (St. Jude Children’s Research Hospital). Human HMG-N1-YFP and HMG-N2-YFP were provided by Dr. Michael Bustin (National Cancer Institute, Bethesda).

Split GFP vectors, pNPSA-Sfi A/B (14)-N157 containing GFP 1–157 aa and pNPSA-Sfi A/B (14)-C158 containing 158–234 aa of GFP were provided by Dr. Paul Duprex (Queen’s University, Belfast). Histone H1 and H2B were PCR amplified with flanking SfiI restriction site to clone into pNPSA-Sfi A/B (14)-N157 vector, which we termed as GFP N-term fused proteins. Similarly, LANA 1–32, 1–32 with 5–15 aa mutated to alanines (mut5), LANA 1–340, LANA-FL and LANA-FL-mut 5 (5–15 aa mutated to alanines) were cloned into pNPSA-Sfi A/B (14)-C158, which we termed as GFP-C term fused proteins.

The KSHV-negative cell line, BJAB and the KSHV-positive cell lines, BCBL1 and JSC1 were cultured in RPMI 1640 medium supplemented with 10% fetal bovine serum, 2 mM L-glutamine, and penicillin-streptomycin (5 U/ml and 5 µg/ml, respectively) as described previously [32], [35], [37]. The human embryonic kidney 293T (HEK293T) cell was Dulbecco's modified Eagle's medium (DMEM) supplemented with 5% FBS, 2 mM L-glutamine, and penicillin-streptomycin (5 U/ml and 5 µg/ml, respectively).

Anti-flag (M2) mouse monoclonal antibody was purchased from Sigma-Aldrich (St. Louis, MO). The anti-Myc (9E10) and anti-HA (12CA5) were purified from hybridoma culture supernatants and purified 9E10 was purchased from Sigma-Aldrich (St. Louis, MO). Anti-LANA mouse monoclonal antibody was obtained from Ke Lan (Institute Pasteur of Shanghai). Rat anti-LANA was purchased from Advanced Biotechnologies (ABI Advanced Biotechnologies, Columbia, MD). Anti-histone H1 Antibody (clone AE-4) and Anti-histone H2B Antibody were purchased from Millipore (EMD Millipore Corporation). Mouse monoclonal anti-histone-H1 was purchased from MBL (MBL Inc.). Anti-GFP antibodies (T-19, cat# sc-5384; GFP01, cat# sc-57587) were purchased from Santa Cruz Biotechnology (Santa Cruz Biotechnology, Inc.).

### Immunoprecipitation and Detection

Cells were lysed in RIPA buffer (50 mM Tris-HCl [pH7.5], 150 mM NaCl, 1 mM EDTA), and 1% NP40 containing protease inhibitors (1 mM phenylmethylsulfonyl fluoride, 10 µg of pepstatin/ml, 10 µg of leupeptin/ml, and 10 µg of aprotinin/ml). Cell debris was removed by centrifuging the cell lysates at high speed. The lysates were incubated with Protein A and G beads for 1 hour at 4°C to pre-clear before incubating with indicated antibodies. The pre-cleared lysates were incubated with specific antibodies overnight at 4°C with constant rotation followed by capturing the immune complex with Protein A and G Sepharose beads. The resulting immunoprecipitates complex were collected by centrifugation at 2,000 × g for 2 min at 4°C. The beads were washed four times with 1 ml RIPA buffer to remove non-specifically attached proteins. The immunoprecipitated proteins were resuspended in 30 µl of SDS sample buffer followed by fractionation on SDS-PAGE and Western transfer using standard protocol (Bio-Rad, Laboratories, Hercules, CA). Specific proteins bands were detected using specific antibodies after incubation with appropriate infrared-dye tagged (IR680, IR800) secondary antibodies and scanning the blot using an Odyssey scanner (Li-Cor Biosciences, Lincoln, NE). Band intensities were determined by an image analysis tool of the Odyssey software (Li-Cor Biosciences, Lincoln, NE).

### DNase I and Micrococcal Nuclease (MNase) Treatments

The lysates for DNase I treatments were supplemented with 5 mM MgSO_4_ and 2 mM CaCl_2_ and incubated with 50 µg DNase I (Sigma) at 4°C for 45 min prior to pre-clearing with Protein A and G. A fraction of DNase I treated and pre-cleared lysate was taken for DNA extractions to PCR amplify the housekeeping gene, GAPDH using specific PCR primers (S-5′-CAGCAAGAGCACAAGAGGAAGA-3′, and AS- 5′-TTGATGGTACATGACAAGGTGCGG-3′). To determine the activity of DNase I, we added 100 ng/µl purified plasmid DNA in the lysates and treated with varying amounts of DNase I followed by extraction of DNA and resolving them on agarose gel. DNase I cleave DNA between nucleosomes but do not disrupt mono-nucleosomes, and limits nonspecific co-precipitations bridged by DNA. For MNase treatments, the lysates were supplemented with 3 mM MgCl_2_ and 1 mM CaCl_2_ and added with 2 U of MNase per reaction (Sigma-Aldrich, St. Louis, MO). The lysate with MNase were incubated for 30 min at 28°C as per manufacturer’s recommendation (Sigma-Aldrich, St. Louis, MO). DNA was extracted from a fraction of the treated lysate to detect the size of DNA mono-nucleosomes by resolving them on agarose gel. MNase cleaves both the double stranded and single stranded nucleic acids DNA and RNA, which generates mono-nucleosomes and therefore limits non-specific interactions bridged by DNA.

### Purification of GST Fusion Proteins

*Escherichia coli* BL21 (DE3) cells were transformed with plasmid containing GST-fusion proteins. Bacterial culture was grown till they reached the optical density (OD_600 nm_) of approximately 0.6. The cultures were induced with 1 mM isopropyl-β-D-thiogalactopyranoside (IPTG) for 4 h at 37°C. The bacteria were pelleted, washed once with 5 ml STE buffer (100 mM NaCl, 10 mM Tris, and 1 mM EDTA, pH 7.5), resuspended in 5 ml NETN buffer (0.5% NP-40, 100 mM NaCl, 20 mM Tris, 1 mM EDTA, pH 8.0) containing protease inhibitors followed by incubating them on ice for 15 min. The suspension was sonicated to solubilize the proteins after adding 75 µl of 1 M dithiothreitol (DTT) and 900 µl of a 10% solution of Sarkosyl in STE buffer. The lysates were centrifuged (13,000×g, 10 min, 4°C) to remove the unsolubilized proteins. The clear supernatant was transferred to fresh tube and added with 1.5 ml of 10% Triton X-100 in STE buffer and 200 µl of Glutathione-Sepharose beads. The tubes were rotated overnight at 4°C to capture the GST-fusion proteins bound to Glutathione-Sepharose beads by washing with NETN buffer containing protease inhibitors. The purity of GST-fusion proteins was determined by SDS-PAGE and densitometric analysis.

### *In-vitro* Translation and Binding Assay

Histone H1 and H2B were *in-vitro* translated using TNT T7 Quick Coupled Transcription-Translation System (Promega Inc. Madison. WI). Briefly, 2 µg of plasmids containing the genes to be translated were used in a 50 µl reaction volume containing 1 mM ^35^S Methionine followed by incubation at 30°C for 90 min. In-vitro translated proteins were incubated overnight at 4°C with GST or GST-fusion proteins in binding buffer (0.1% NP-40, 0.5 mM dithiothreitol [DTT], and 10% glycerol in 1X phosphate-buffered saline [PBS] containing protease and phosphatase inhibitors). Loosely bound proteins were removed by washing the complex three times with binding buffer containing 250 mM of NaCl. Bound Samples were mixed with SDS-sample buffer, incubated at 90°C for 3 min, and resolved on SDS-PAGE. The gels were dried and exposed to a Phosphorimager plate to determine the fraction of bound proteins by scanning on a Typhoon 9410 Phosphorimager (Molecular Dynamics, Inc., Sunnyvale, CA).

For binding of eukaryotically expressed proteins with GST fusion proteins, cells expressing target proteins were lysed in RIPA buffer, centrifuged to remove cell debris before pre-cleaning with GST-alone by rotating at 4°C for 3 h. In the case of DNase I treatment, cell lysate was treated with 50 µg of DNase I as described above before pre-clearing with GST-alone beads. GST-bound complex was washed three times with RIPA buffer containing 250 mM of NaCl to remove any loosely bound proteins. Bound proteins were mixed with SDS-PAGE loading dye, incubated at 90°C for 3 min before resolving them on SDS-PAGE and detecting the target proteins on a Western blot using specific antibody scanned by a LiCor Imager (Li-Cor Biosciences, Lincoln, NE).

### Immunofluorescence Assay

Cells were washed once with 1XPBS and spread evenly on a cover slips before air-drying. The cells were fixed for 10 min at room temperature with 4% paraformaldehyde followed by permeabilization with 0.2% Triton X-100 in PBS for 10 min. Cells were subjected for blocking the non-specific targets by incubating with 0.4% fish skin gelatin and 0.05% Triton X-100 in 1XPBS. Blocked cells were then incubated with primary antibodies for overnight at 4°C under moist conditions. The primary antibodies were removed by washing the cells three times with 1XPBS containing 0.4% fish skin gelatin (Sigma-Aldrich, St Louis, MO) and 0.05% Triton X-100 followed by incubating the cells with Alexa Fluor conjugated secondary antibodies (Invitrogen, Inc. Carlsbad, CA) at room temperature for 1 h. Cells were washed with 1XPBS containing 0.4% fish skin gelatin and 0.05% Triton X-100 three times to remove unbound antibodies followed by counterstaining with DAPI (4′, 6′-diamidino-2-phenylindole). The cells were examined using a confocal microscope equipped with Fluoview FV300 (Olympus Inc., Melville, NY). The captured images were analyzed with Flouview Image analysis software (Olympus Inc., Melville, NY).

### Preparation of Chromosome Spreads and Immune Localization

Mitotic phase cells were obtained by treating them with colcemid (0.1 µg/ml) at 37°C for 1 h before preparing the mitotic chromosome spreads. Treated cells were washed with 1XPBS followed by pelleting them with centrifugation and resuspending in RSB buffer (10 mM Tris [pH 7.4], 10 mM NaCl, 5 mM MgCl_2_) to a final cell concentration of 3X10^6^/ml. Cell suspensions were incubated at 37°C for 10 min followed by transferring them onto ice. Cells were dropped onto positively charged poly-L-lysine-coated coverslips to break open cells. Air-dried cover slips were treated with 4% paraformaldehyde to fix the chromosomes followed by treating with 0.5% Triton X-100-PBS buffer. Cells were incubated with 1XPBS containing 0.4% fish skin gelatin and 0.05% Triton X-100. Cells were then incubated with primary antibodies under moist conditions for overnight at followed by washing them with 1XPBS containing 0.4% fish skin gelatin and 0.05% Triton X-100. The cover clips were incubated with Alexa Fluor conjugated secondary antibodies (Molecular Probes) at room temperature for 1 h to followed by washing them with 1XPBS containing 0.4% fish skin gelatin and 0.05% Triton X-100 three times to remove non-specifically bound antibodies. The chromosomes were visualized by counter-staining the coverslip with DAPI (4′, 6′-diamidino-2-phenylindole). The images were captured with a Fluoview FV300 (Olympus Inc., Melville, NY) confocal microscope followed by analyzing them with FLUOVIEW image analysis software (Olympus Inc., Melville, NY).

### Production of Lentivirus and Transduction of KSHV-infected Cells

Lentiviruses containing gene of interest were produced by transient transfection of lentiviral vector along with vectors to express packaging proteins into HEK 293T cells as described previously [38]. Briefly, cells were seeded in 100 mm dishes in DMEM (HyClone, Logan, UT) supplemented with 10% FBS and 1% antibiotic-antimycotic. 60% confluent cells were transfected with a total of 20 µg of plasmid DNA containing 1.5 µg of envelope plasmid pCMV-VSV-G, 3 µg of packaging plasmid pRSV-Rev, 5 µg of packaging plasmid pMDLg/pRRE, and 10.5 µg of lentiviral vector containing gene of interest using calcium phosphate transfection. Transfected cells were induced with sodium butyrate by changing the medium containing 10 mM HEPES, and 10 mM sodium butyrate. The viruses in the supernatant were collected in DMEM containing 10 mM HEPES at every 12 h for 2 days. Supernatant containing viruses were centrifuged at 4000×g for 10 min at 4°C to remove cell debris followed by filtering the supernatant through 0.45-µm-pore-size membrane filters. The viruses were further concentrated by centrifuging at 70,000 × g for 2 h at 4°C. BCBL1, JSC1 and BJAB cells were plated at 10^5^ cells in six-well plate and transduced with virions in the presence of polybrene (8 mg/ml). Transduced cells were selected with 2 µg/ml puromycin to eliminate un-transduced cells. Transduced cells were used for the analysis of proteins in a Western blot and chromosome spreads after obtaining 100% GFP fluorescence.

### GFP Complementation Assay

Plasmids containing N-term GFP (1–157 aa) fused to histone H1 or histone H2B and plasmids containing GFP-C-term (158–238 aa) fused LANA truncations and mutants were transfected in various combinations into 293T cells by electroporation. Transfected cells were plated on 100 mm culture dish as well as onto cover slips to visualize GFP signal under the fluorescent microscope. 48 h post-transfection, cells were observed and imaged for fluorescence using inverted microscope, Olympus IX60 (Olympus Inc., Melville, NY).

### Fluorescence Resonance Energy Transfer (FRET) Assay

FRET by sensitized emission of the acceptor fluorophore was performed as described previously [39]. Briefly, fluorescence resonance energy transfer (FRET), between a pair of fluorophores, is an indication of closer proximity than colocalization and is used to study in-vivo protein-protein interaction by using fluorescently labeled proteins. FRET technique presents a method for detection of energy transfer from a donor fluorophore to an acceptor fluorophore after confocal sensitized emission. We used a widely used FRET pair of Cyan Fluorescent Protein (CFP) as donor and Yellow Fluorescent Protein (YFP) as acceptor. Histones, H1 as well ad H2B were fused with CFP and LANA was fused with YFP. 293T cells transduced with CFP-fused histones were transfected with YFP-LANA for normalization and capturing images for FRET analysis. FRET index was determined by previously utilized method [39].

### Yeast-2-hybrid and ONPG Assay

Amino terminal domain of LANA (1–340 aa) was PCR amplified to clone in-frame with GAL4-DBD (DNA binding domain) as bait in vector, pAS1 (Clontech, Mountain View, CA). Various LANA interacting proteins, histone H1, H2B, MeCP2, DEK, HMG-N1 and HMG-N2 were cloned in-frame with GAL4-ACT domain as prey in vector, pACT (Clontech, Mountain View, CA). Sequences of both the bait and prey fused to GAL4-DBD and ACT domains, respectively, were confirmed by sequencing. LANA bait was transformed into *S. cerevesiae* strain Y190 (Clontech, Mountain View, CA) and selected on a DOBA-Trp dropout medium (Clontech, Mountain View, CA). Expression of LANA-DBD was confirmed on a Western blot by using anti-GAL4 DBD antibody (Santa Cruz Biotechnology Inc. Dallas, TX). Y190 containing LANA 1–340 aa-DBD was transformed with pACT containing different prey and selected on BOBA-Trp, His, Leu dropout medium (Clontech, Mountain View, CA) in the presence of 50 mM 3-aminotriazole as described previously [40]. Selected colonies were His^−^ medium in the presence of 50 mM 3-aminotriazole followed by selection for induction of beta-galactosidase activity. Subsequent interaction assays were performed by measuring the beta-galactosidase activity using 2-nitrophenol-β-D-galactopyranoside as substrate on three independent co-transformants. The amount of released 2-nitrophenol due to beta-galactosidase was measured by recording the absorbance at 420 nm.

## Results

KSHV encoded LANA is a large nuclear protein required for tethering of viral episomes to the host chromosome in order to retain its genome through various cell divisions. LANA has been shown to interact with a large number of host’s chromatin molecules, which help in tethering of LANA to the host chromosome and most likely in passaging of the viral episomes to the dividing tumor cells [2], [3], [17], [41]. Binding of LANA to various cellular targets has been debated over the years therefore we wanted to comprehensively analyze its binding with histones using a variety of biochemical assays.

### LANA Binds to Core as well as Linker Histones

**Figure 1:**
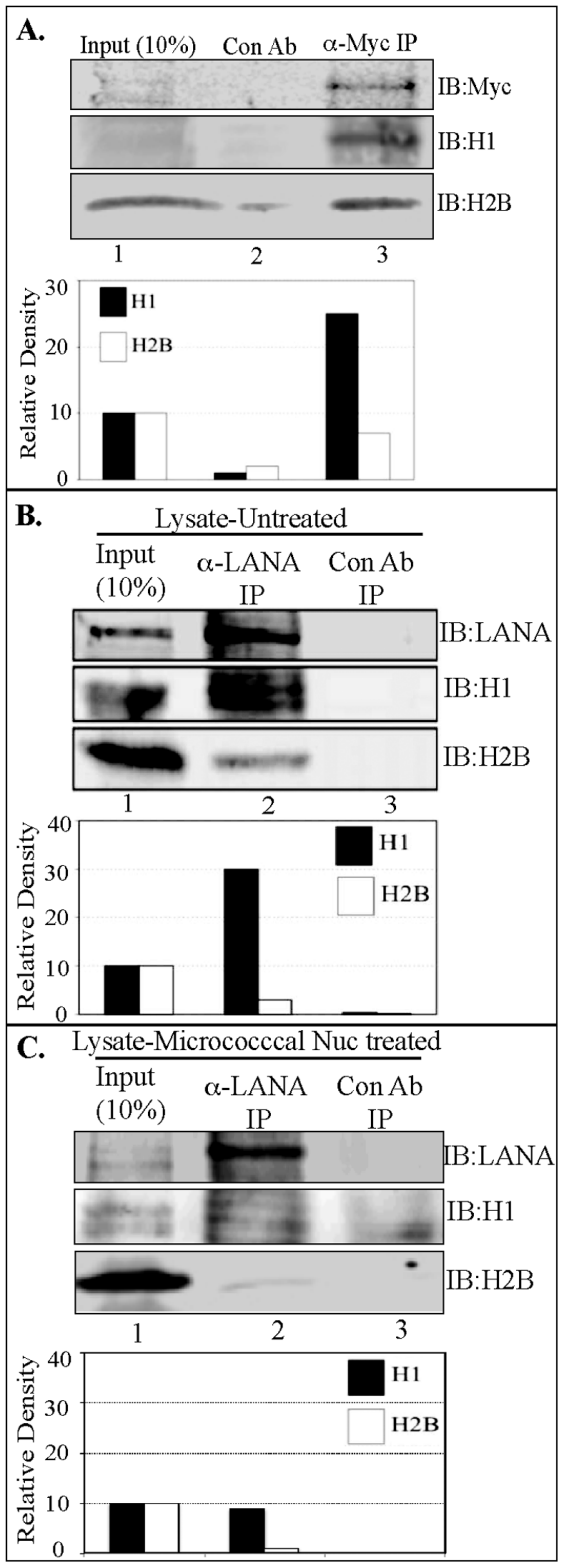
LANA binds to histones H2B and histone H1. **A.** *In-vitro* translated LANA binds to purified histone H1 and H2B. Myc epitope tagged LANA was in-vitro translated and incubated with purified histone containing linker as well as core histones. Immunoprecipitation of LANA with anti-myc antibody and immunoblot (IB) using anti-histone H1 and anti-H2B showed binding with LANA (lane 3). Relative binding of histone H1 and H2B were determined by setting the intensity of input band (lane 1) at 10% and calculating the relative densities of based on band intensity of input lanes. Isotype control antibody did not show any delectable binding (lane 2). **B.** 30 millions BCBL1 cells were lysed in RIPA buffer to immunoprecipitate LANA using anti-LANA antibody and detection of co-precipitated histone H1 and H2B. Immunoblot (IB) with anti-H1 and anti-H2B showed co-immunoprecipitation of both the histones (lane 2). Isotype control antibody did not now any detectable binding (lane 3). Relative binding of histone H1 and H2B were determined by setting the densities of input lanes (lane 1) at 10% and calculating the relative densities of immunoprecipitated bands in lane 2. **C.** Lysates of BCBL1 from 30 million cells were treated with 2 U of micrococcal nuclease (MNase) to digest the inter-nucleosomal DNA for generating mononucleosomes. MNase treated lysates was subjected for immunoprecipitation with anti-LANA antibody. Immune detection of histone H2B showed reduced level of H2B as compared to H1 (lane 2). Relative density calculated by using input as 10% showed reduced binding of histone H1 (approx. 10%) as compared to 30% without MNase treatment.

The first nucleosomal molecule shown to bind with LANA was linker histone H1 [2], however, additional studies identified LANA’s interaction with core histones, H2B/H2A [20]. Here, we revisited the binding of LANA with histones using a variety of biochemical in-vitro and in-vivo assays. In-vitro-translated myc tagged LANA incubated with purified histones from calf thymus (Sigma, cat #H9250) followed by immunoprecipitation with anti-myc antibody was able to co-immunoprecipitate histone H1 as well as H2B (Fig. 1A). Importantly, the relative binding of H1 was higher than H2B when compared to the amounts present in respective input lanes (Fig. 1A, relative density). The earlier study had tested the binding of LANA with histone H1 and H3 but not with H2B [2]. The binding of LANA with histones was further evaluated from the cell lysates of KSHV infected primary effusion lymphoma cells, BCBL1. Immunoprecipitation of LANA with anti-LANA rat monoclonal antibody efficiently precipitated both the histones H1 and H2B. However, the relative binding to H2B was lower than H1, similar to the purified histone binding data (Fig. 1, A and B). Treatment of the lysate with micrococcal nuclease, which cleaves the nucleic acid between the nucleosomes and generates mononucleosomes, significantly reduced H2B binding to LANA (Fig. 1C, lane 2). Importantly, MNase treatment showed reduction of LANA association with histone H1 but was still higher than H2B (Fig. 1C). Immunoprecipitations with isotype control antibody did not show binding of histones confirming the specificity of the assay. Generation of mono-nucleosomes was confirmed by treating the cell lysates with different concentrations of MNase followed by extraction of DNA and resolving them on an Agarose gel. A representative gel with DNA (147 bp) from a single nucleosome is shown in Figure S1. Binding of LANA with histones was also tested in other PEL cell line, JSC1, which showed binding with both the histones, H1 and H2B (data not shown).

### LANA 1–32 Stably Expressed in BJAB Cells Binds to Histone H2B

**Figure 2:**
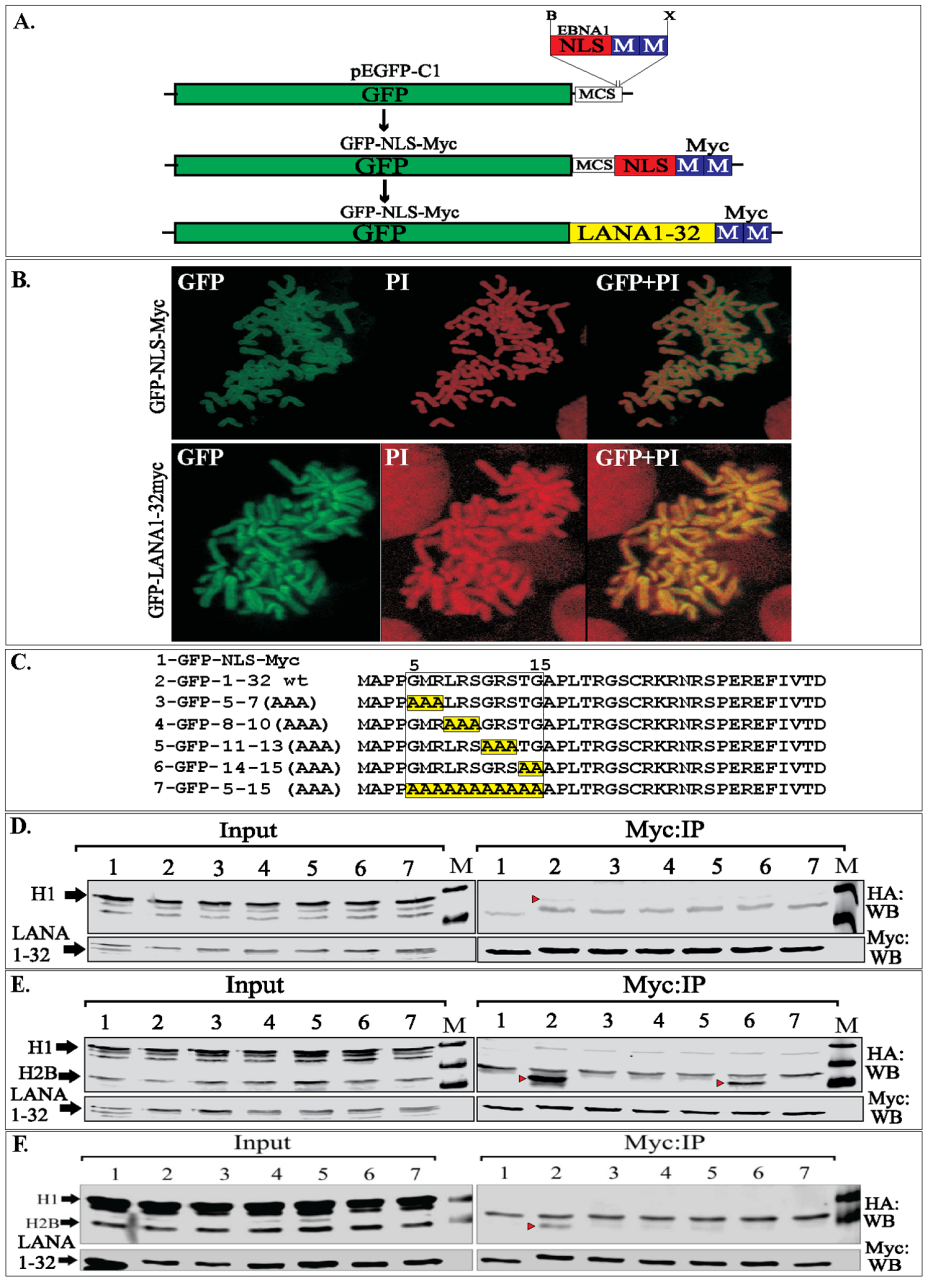
Stably expressing LANA 1–32 aa polypeptide bond to histone H2B. **A.** Schematic showing the strategy for generating GFP-NLS myc. Oligo containing a Nuclear Localization Sequence (NLS) of EBNA1 with two-tandem myc tag epitope was cloned at BamHI and XbaI sites (MCS) of pEFGCP-C1 vector to generate GFP fused with NLS and myc tag (GFP-NLS-myc). LANA 1–32 aa was PCR amplified with primers flanked with EcoRI and BamHI sites on the 5′ and 3′ respectively. GFP-NLS-myc was digested with EcoRI and BamHI, which released EBNA1 NLS, to clone LANA 1–32 aa (GFP-1–32 aa-myc). **B.** BJAB stably expressing GFP-NLS-myc or GFP-LANA 1–32myc was subjected for chromosome spreads and the nuclei were stained with propidium iodide (PI). GFP-LANA1–32myc completely painted the chromosomes whereas GFP-NLS-myc localized to nucleus but did not stain the chromosome. **C.** LANA 1–32 aa sequence showing CBD (5–15 aa) and its alanine substitution mutants highlighted in yellow. **D.** Histone H1 tagged with HA were transfected with GFP-NLS-myc (control) (lane 1) and GFP-LANA1–32 aa with wt CBD (lane 2) and its alanine substitution mutants (lanes 3–7 corresponding to mutants 3–7 in panel C. WB blot with anti-HA antibody showed a band of H1 with wt CBD LANA indicated with red triangle in the myc IP panel. Input showed uniformed expression of histone H1 in the input lanes. GFP-NLS-myc or GFP-LANA 1–32 and its mutants were detected with anti-myc in the input as well as IP panels. M shows the protein marker lane. Non-specific signals were detected below the red triangle in HA:WB panel. **E.** HA tagged histone H1 and H2B were co-transfected with GFP-NLS-myc (lane 1) or GFP-LANA1–32 wt (lane 2) and CBD mutants (lanes 3–7 corresponding to the mutants in panel C). Precipitation of GFP-NLS-myc and LANA 1–32 and it mutants showed co-precipitation of H2B (indicated with red triangle) with wt CBD containing LANA 1–32 (lane 2) and relatively lower amount with mutant 14-TG-15 (lane 6). GFP-NLS-myc and GFP-LANA1–32 and its mutants were detected with anti-myc blot in input as well as myc:IP lanes. IgG light chain was detected in HA:WB panel. **F.** Cells co-transfected with H1-HA and H2B-HA and GFP-NLS-myc or GFP-LANA1–32 aa and its mutants were lysed and the lysates were treated with 50 ug of DNase I for 45 min before immunoprecipitation. Co-precipitating H2B was detected in LANA 1–32 aa with wt CBD (lane 2). Both histones expressed in all the lanes detected by anti-HA WB. GFP-NLS-myc and LANA1–32 along with its mutants were detected with anti-myc WB. IgG light chain was detected in HA:WB panel above the H2B specific band.

We generated a BJAB cell line stably expressing GFP fused to nuclear localization signals (NLS) of Epstein-Bar Virus (EBV) encoded EBNA1 and two tandem myc tag for the immunoprecipitations with specific monoclonal anti-myc antibody. Introduction of NLS was to ensure that GFP localizes to the nucleus, as shown before [20] and thus serves as a more stringent control to LANA 1–32 aa, which contains nuclear localization signals (Fig. 2A). Chromosome spreads of these stable cells were prepared to visualize the localization of GFP and GFP fused LANA 1–32 aa on the chromosomes. GFP-NLS localized onto the chromosomes but was unable to stain the chromosome uniformly like GFP-LANA1–32 aa, which showed yellow signals in the merge panel with propidium iodide (PI) stained chromatin (Fig. 2B). This confirmed that LANA 1–32 aa has the ability to bind with proteins on the host chromosome as shown before [20]. Immunoprecipitation of GFP and GFP-LANA1–32 aa with anti-myc antibody showed immunoprecipitation of histone H2B only with LANA 1–32 aa as detected before [20].

### Histone H1 Binds to LANA 1–32 aa with Relatively Low Affinity

We used an expression system in which histone H1 and H2B were exogenously expressed with LANA 1–32 aa to determine its relative binding with histones. We also used previously described [20] alanine mutants of chromosome binding domain (CBD), 5–15 aa for binding with H2B to ensure the specificity of our assay. We also used histone H1 to test its binding with LANA 1–32. Immunoprecipitation with anti-myc antibody to precipitate GFP-NLS-myc (control) and LANA or its alanine mutants showed a rather weak signal for histone H1 co-immunoprecipitating with wt LANA 1–32 aa but not with any other mutants (Fig. 2D, compare lane 2 with 3–7). However, when histone H1 was co-expressed with histone H2B, LANA 1–32 aa was not able to precipitate detectable levels of histone H1 (Fig. 2E). Not surprisingly, LANA 1–32 aa precipitated histone H2B as shown previously (Fig. 2E) [20]. Among the alanine substitutions mutants, LANA 1–32 aa 14–15 (TG) precipitated histone H2B at reduced levels as compared to wt-LANA 1–32 aa (Fig. 2D, compare lanes 2 and 6) similar to previous reports [20]. Treating the lysates with DNase I to cleave the inter-nucleosomal DNA of cells co-expressing histone H1 and H2B with LANA 1–32 aa and its CBD alanine substitution mutants showed precipitation of H2B with only wt-LANA 1–32 aa, although at a much reduced level (Fig. 2F).

We further explored the association of endogenous histones with LANA 1–32 aa and its alanine substitution mutants by transfecting them into BJAB cells and immunoprecipitating with anti-myc antibody to precipitate LANA 1–32 aa and its mutants and co-detecting histone H1 and H2B with specific antibodies. Similar to the above expression assay, LANA 1–32 aa was able to precipitate low levels of histone H1 but significantly higher levels of histone H2B when co-expressed during transient transfection assays (Fig. S2, compare lane 1 containing wt LANA 1–32 aa with its alanine mutants in lanes, 2–5).

### LANA 1–340 aa Binds to Histone H2B as well as H1

**Figure 3:**
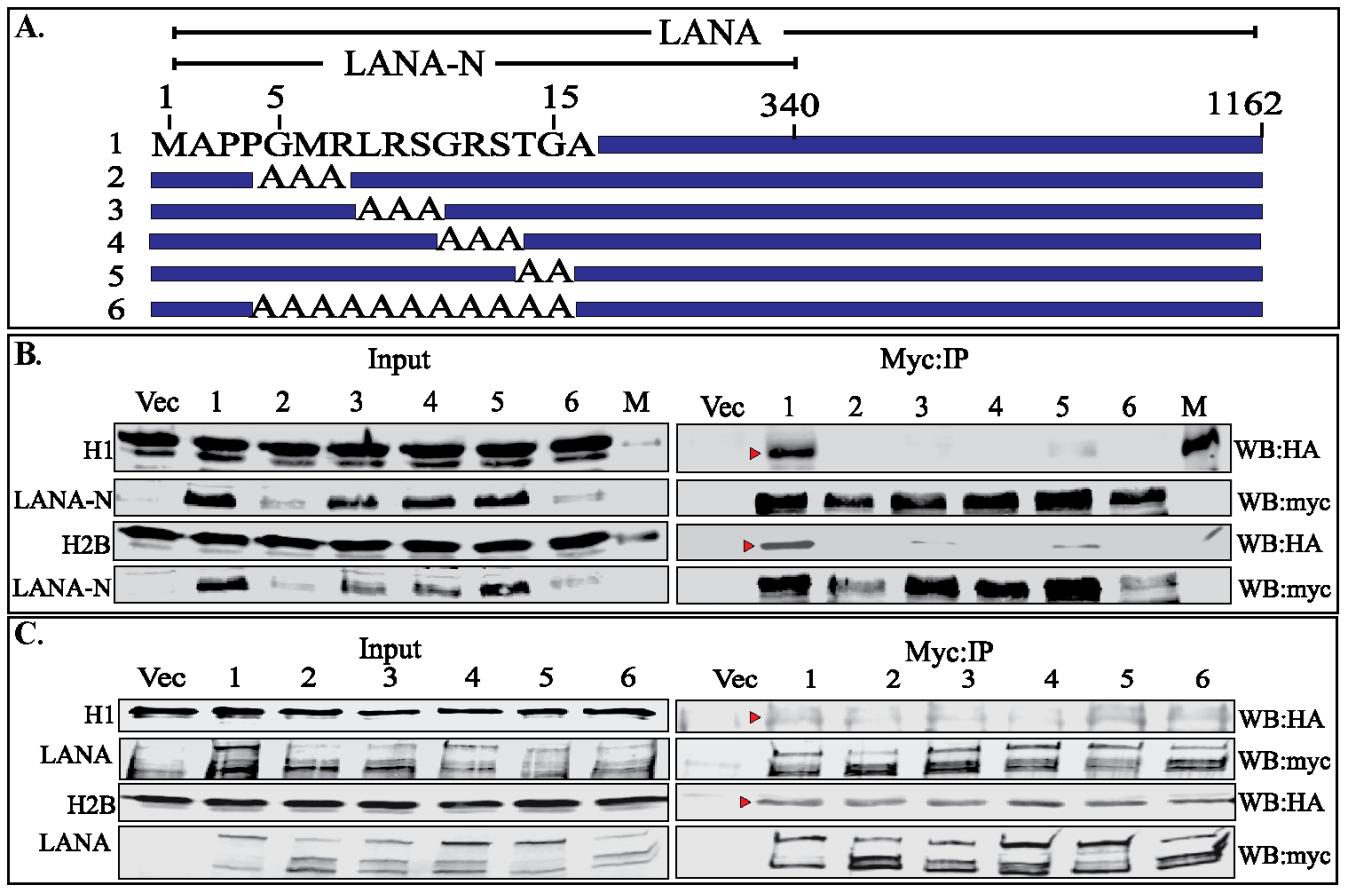
The LANA-FL and 1–340 aa polypeptide precipitated histone H1 as well as H2B. **A.** Schematic of LANA 1–340 aa with marked CBD (5–15 aa). Mutants 2–6 were alanine substitution mutants of CBD. **B.** HA tagged histone H1 or H2B was co-transfected with myc vector (Vec) or LANA 1–340 aa-myc (lane 1) and its CBD mutants (lanes 2–6). Lane M was the marker and lane Vec contain HA-H1 or H2B co-transfected with myc vector. LANA1–340 aa and its alanine mutants were immunoprecipitated with anti-myc antibody and detected with myc antibody (WB:myc). Histones, H1 and H2B in the input and IP lanes were detected with anti-HA antibody (WB:HA). LANA1–340 aa with wt CBD (lane 1) co-precipitated histone H1 and a small amount with CBD mutant 14-TG-15 (lane 5). Histone H2B was co-precipitated with LANA 1–340 aa with wt CBD (lane 1) as well as alanine mutants in lane 3, 4, 5 and small amount in lane 6. **C.** HA tagged histone H1 or H2B was co-transfected with myc vector (Vec) or LANA-FL-myc (lane 1) and its CBD mutants (lanes 2–6). LANA and its alanine mutants were immunoprecipitated with anti-myc antibody and detected with myc antibody (WB:myc). Histones, H1 and H2B in the input and IP lanes were detected with anti-HA antibody (WB:HA). LANA with wt CBD (lane 1) as well as all the alanine mutants (lanes, 2–6) co-precipitated histone H1. Histone H2B was co-precipitated with LANA containing wt CBD and all the alanine substitution mutants (lanes 2–6).

We further wanted to determine whether increasing the length of the LANA bait from 1–32 aa to N-term (1–340 aa) may have an effect on binding with the histones. Increasing the length of the bait may change the overall conformation of the molecule. In order to test this hypothesis, we expressed LANA 1–340 aa-myc and its alanine substitution mutants (Fig. 3A) with either histone H1 or H2B. LANA 1–340 aa and its mutants were immunoprecipitated with anti-myc antibody, which showed co-precipitation of histone H1 with wild type LANA (Fig. 3B, lane 2) and very low levels with 14–15 aa (TG) mutant (Fig. 3B, compare lanes 1, and 5). Histone H2B was efficiently precipitated with LANA 1–340 aa with wt CBD (5–15 aa) but was significantly reduced or absent in mutants with alanine substitutions (Fig. 3B, lanes 2–6). Interestingly, when both the histones, H1 and H2B were co-expressed together with LANA 1–340 aa and its alanine mutants, the levels of histone H1 co-precipitating with LANA 1–340 aa were significantly reduced (Data not shown).

### LANA-FL Associates with Histone H1 when Expressed in Human Cells

We further analyzed the bindings of histone H1 and H2B by co-expressing full length LANA and alanine substitution mutants within the chromatin-binding domain (CBD), 5–15 aa of LANA (Fig. 3A) along with individual histones (Fig. 3C). Individually expressed histone H1 with LANA-FL showed binding with LANA containing wt CBD as well as its alanine substitution mutants. Immunoprecipitated LANA and its alanine substitution mutants were detected with anti-myc antibody (Fig. 3C). Similarly, H2B co-precipitated with LANA very efficiently and the levels of H2B bound to LANA did not significantly change with the introduction of alanines in the CBD (Fig. 3C, compare lanes 1–6) suggesting that histone H2B can also bind to other domains of LANA. Since LANA with wild type CBD showed faint binding with histone H1, we wanted to further confirm their binding by using histones fused with another epitope tag (Flag-tag). Flag-tagged histone H1 or H2B were co-expressed with LANA-myc or with myc vector to immunoprecipitate LANA. Detection of H1 and H2B with anti-Flag antibody showed co-immunoprecipitation of both histones (Fig. S3A, IB:HA). DNase I treatment of the lysate was unable to significantly reduce the binding of H1 or H2B suggesting that these proteins may bind to LANA independently (Fig. S3B). In order to compare the levels of exogenously expressed histones with endogenous protein, these blots were incubated with histone H1 and H2B specific antibodies, which showed slightly higher levels of over-expressed proteins (Fig. S3A and B). Importantly, endogenous histones, H1 and H2B were precipitated with LANA with, as well as without DNase I treated lysates (Fig. S3A and B). Since the intensities of HA tagged H1 and H2B in the immunoprecipitated lanes were faint, we confirmed the specificity of these bands by analyzing cells without exogenous expression of histones. Lack of anti-HA signals in vector or LANA expressing cells confirmed the specificity of H1-HA and H2BA bands (Fig. S3C). Detection of endogenous histones using specific antibodies showed immunoprecipitation of both H1 and H2B with LANA (Fig. S3C).

We also performed reverse immunoprecipitation in which LANA with wt CBD or its alanine substitution mutants co-expressed with either histone H1 or H2B were subjected to immunoprecipitation for histones followed by detection of co-precipitating LANAs. We showed that histone H1 was unable to precipitate detectable levels of LANA or CBD mutants (Fig. S4B). The levels of immunoprecipitating histone H1 were detected with anti-Flag antibody (Fig. S4B). In contrast, histone H2B was able to precipitate LANA as well as alanine substitution mutants (Fig. S4C). H2B-flag in the inputs as well as anti-Flag IP lanes were detected by anti-Flag blot (Fig. S4B). These data suggest that histone H2B binds to LANA with higher affinity compared to H1.

### LANA 1–340 aa and Full-length LANA Immunoprecipitated Endogenous Histones

**Figure 4:**
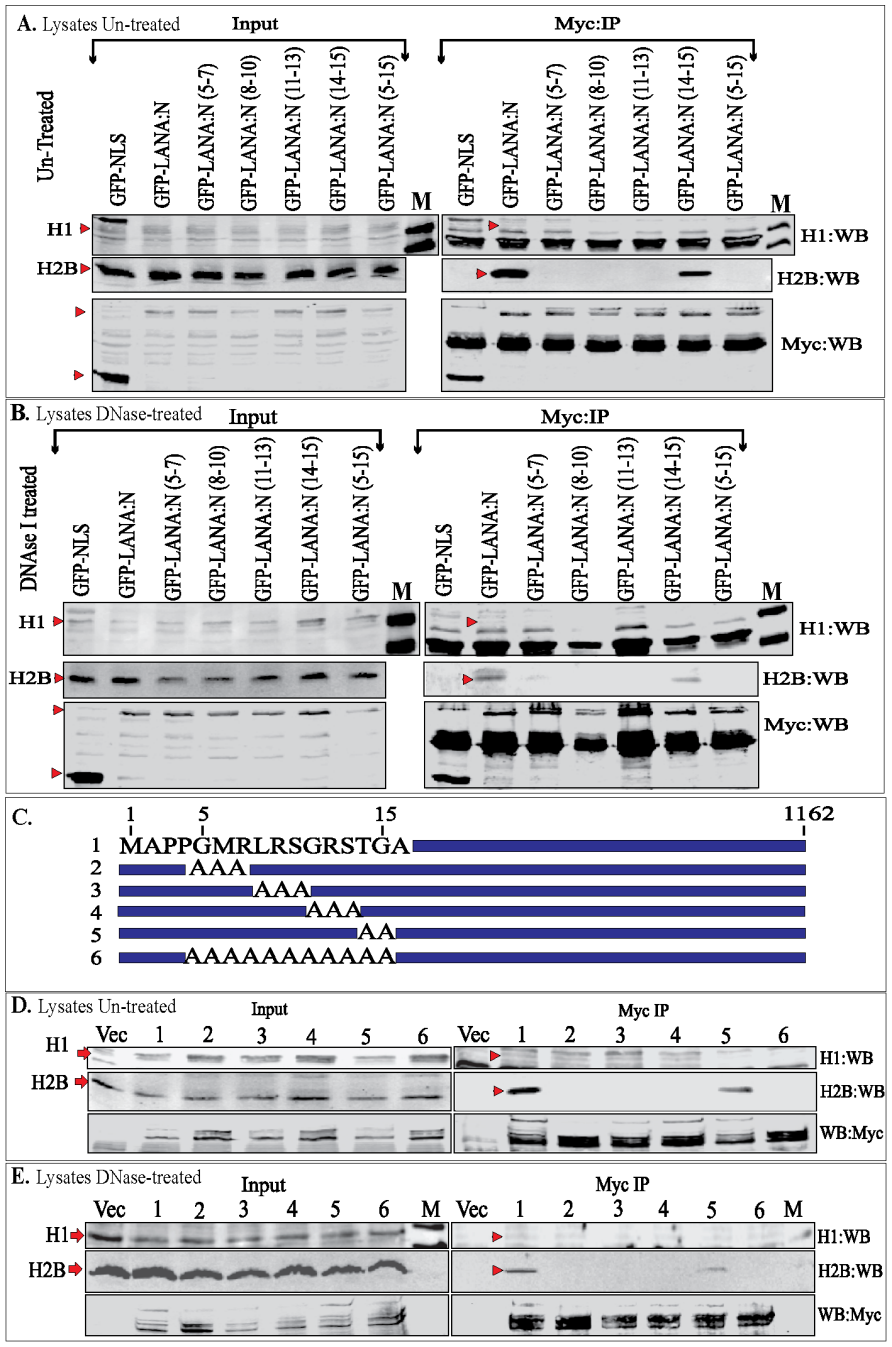
Immunoprecipitation of endogenous histones with LANA-N and LANA-FL. **A.** 293T cells were transfected with GFP-NLS-myc (control) or GFP-LANA-N (1–340 aa)-myc or its alanine substitution mutants (5–7 aa to alanine) and other respective mutants. Lysates were subjected to anti-myc IP without any treatment and immunoprecipitating GFP fusion proteins were detected with anti-myc antibody (Myc:WB panel, red triangle). Endogenous levels of histones were detected using anti-histone H1 (H1:WB) and anti-H2B (H2B:WB) antibodies (indicated red triangle). IgG light chain was detected in H1:WB panel in all the lanes. **B.** Lysate from 293T cells transfected with GFP-NLS-myc (control) or GFP-LANA-N and its alanine mutants were treated with 45 ug DNase I for 45 min before immunoprecipitation with anti-myc antibody. Histone H1 and H2B were detected with specific antibodies (red triangle). IgG light chain was detected in H1:WB panel in all the lanes. **C.** Schematic of LANA-FL with marked CBD (5–15 aa). Mutants 2–6 were alanine substitution mutants of CBD. **D.** 293 T cells were transfected with myc vector (lane Vec) or myc tagged LANA-FL (lane 1) and its alanine substitution mutants (lanes 2–6). Cell lysate from the transfected cells were subjected for immunoprecipitation with anti-myc antibody followed detection of LANA and its mutants in anti-myc WB (WB:myc). Histone H1 and histone H2B were detected with specific antibodies (red triangle). **E.** Cells transfected with above plasmid were lysed and the lysates were treated with 45 ug of DNase I before immunoprecipitation with anti-myc antibody. Histone H1 and H2B were detected using specific antibodies (red triangle).

We further investigated whether amino terminal domains of LANA (1–340 aa) and full length LANA can immunoprecipitate endogenous histone H1 and H2B. We transfected LANA 1–340 aa and alanine substitution mutants of CBD fused to GFP and myc tag into 293 cells. GFP-NLS-myc was used as a control in this experiment. Precipitation of amino terminus-LANA with anti-myc antibody and detection of both histone H1 and H2B using specific antibodies showed a distinct precipitation of H2B with wt LANA as well as alanine substitution mutant 14-TG-15 aa (Fig. 4A), similar to H2B binding with LANA 1–32 aa. In contrast, histone H1 showed a very faint band in LANA-N lane with wt CBD, although the reactivity of anti-histone H1 antibody was significantly lower as compared to anti-H2B antibody (Fig. 4A, H1 and H2B in input lanes are identified with red arrow). GFP-NLS-myc and GFP-LANA 1–340 aa-myc were detected using anti-myc WB (Fig. 4A). We also treated the lysates with DNase I to cleave inter-nucleosomal DNA, which may indirectly link the histones. Immune detection of histone H1 and H2B in the precipitation complex with LANA-N with wt CBD and alanine substitution mutants detected a faint signal of H1 in the wt CBD and mutant 11–13, suggesting that histone H1 can directly bind to LANA (Fig. 4B). Histone H2B was similar to without DNase I treatment and showed binding to LANA with wt CBD and mutant 14-TG-15 aa (Fig. 4B).

We also determined whether full length LANA was able to precipitate endogenous histones, H1 and H2B under DNase I untreated and treated conditions. LANA-myc with wt CBD and alanine mutants in the CBD were transfected into 293T cells and immunoprecipitated with anti-myc antibody to detect histones using specific antibodies. LANA-FL similar to the amino-terminal domain of LANA (Fig. 4D and E) precipitated histone H1 at relatively low levels but their association was not abolished by DNase I treatment (Fig. 4D and E). Immune detection of histone H2B showed that LANA with wt CBD and mutant 14-TG-15 aa precipitated H2B similar to LANA 1–32 aa and 1–340 aa and their association was still detected after DNase I treatment (Fig. 4 D and E). Levels of LANA with wt CBD and its alanine mutants in the input and IP lanes were detected with anti-myc antibody (Fig. 4 D and E). These data suggest that endogenous histones, H1 as well as H2B can bind to LANA, although H2B bound with a relatively higher affinity, and were independent of each other.

### LANA 1–32 Interacts with Histone H1 and H2B in-vitro

**Figure 5:**
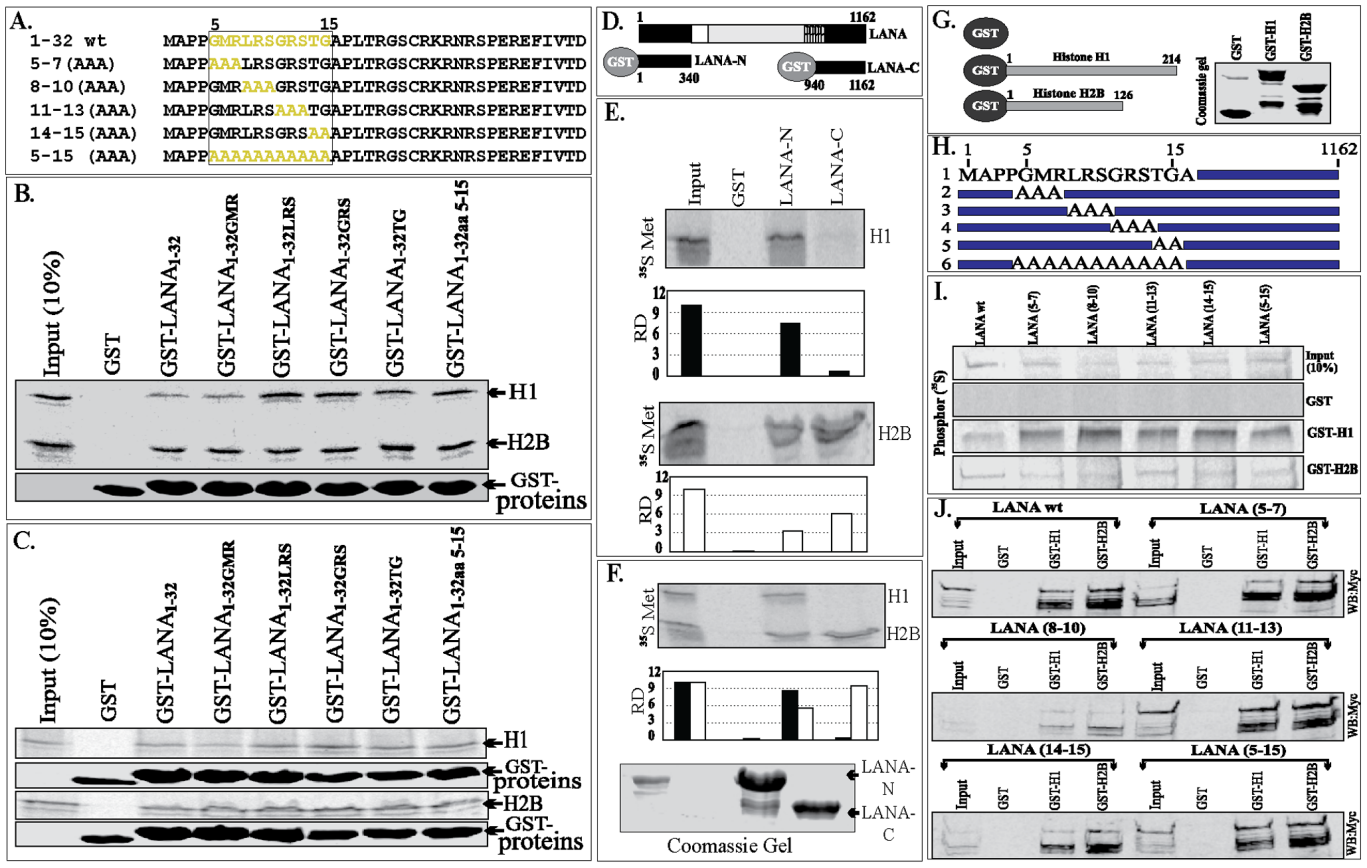
In-vitro binding of LANA with histones, H1 and H2B. **A.** Amino acid sequence of LANA 1–32 aa with CBD sequences in yellow. Alanine substitution mutants of CBD are shown in yellow. **B.** In-vitro translated histone H1 or H2B were incubated with GST (control) or GST-LANA1–32 and its alanine substitution mutants. Bound fraction of H1 and H2B were detected by 35S methionine label on H1 and H2B. Amounts of GST and LANA1–32 aa GST-fusion proteins used in the binding assay are shown in respective GST-protein panels. **C.** Histone H1 and H2B expressed in 293T cells were subjected for binding with GST (control), GST-LANA1–32 aa and CBD mutants. Bound histones were detected in a western blot with anti-HA antibody. GST proteins used in the binding assay are shown in GST-panels. **D.** LANA schematic showing the regions of LANA used as N-term (1–340 aa) and C-term (940–1162 aa). **E.** ^35^S methionine labeled histone H1 and H2B were in-vitro translated for binding with GST (control), GST-LANA-N and GST-LANA-C fusion proteins. Relative densities (RD) of bound histone H1 (black bar) and H2B (white bar) with LANA-N and LANA-C were determined by comparing with respective inputs at 10%. **F.** In-vitro translated histone H1 and H2B were mixed before binding with GST fusion proteins. Relative binding of both the histones with LANA-N and C were determined by the densities of the bands in respective lanes. Commassie stained gel of GST-LANA-N and GST-LANA-C used in the assay. **G**. Schematic to show GST (control) and GST-H1 and GST-H2B fusion proteins. **H**. Schematic of LANA-FL with wt CBD (1) and its alanine mutants from 2–6. **I.** LANA and its alanine substitution mutants were in-vitro translated with ^35^S methionine for binding with histone H1 and H2B. In-vitro translated products were subjected for binding with GST (control), GST-H1 and GST-H2B. The bound fractions were determined, which showed efficient binding of wt LANA as well as alanine mutants to histones, H1 and H2B. **J.** LANA with wt CBD and its alanine substitution mutants were expressed in 293T cells. Lysates were subjected for binding with GST (control), GST-H1 and GST-H2B. Bound fraction were detected with anti-myc WB, which showed binding of wt LANA as well as its mutants to both histone H1 and H2B but not to control GST.

Although the lysates were treated with DNase I and MNase to cleave any intercalating nucleic acids between proteins in the above experiments, we further wanted to determine whether LANA could directly bind to linker and core histones. In-vitro translated histone H1 or H2B were incubated with GST fused LANA 1–32 aa containing wt CBD or its alanine mutants, which bound to both the histones, H1 and H2B (Fig. 5B). The binding reactions were washed with buffer containing 300 mM NaCl to increase the specificity, but we still detected the binding of H2B with wt CBD as well as other alanine mutants (Fig. 5B). Control GST protein did not show any binding with either H1 or H2B suggesting that the binding was not due GST portion of the GST-LANA fusion proteins (Fig. 5B).

We also wanted to determine whether eukaryotically expressed histone H1 and H2B have different affinities to LANA 1–32 due to post-translational modifications as compared to in-vitro translated H1 and H2B. We transfected H1 and H2B expressing plasmids into 293T cells and the lysates were divided equally to bind with control GST or GST fusion of LANA 1–32 aa and alanine substitution mutants followed by washing the buffer containing 300 mM NaCl (Fig. 5A). Detection of H1 and H2B in Western blot showed precipitation of both histone H1 and H2B with LANA 1–32 aa containing wt or alanine mutants similar to the in-vitro translated histones (Fig. 5C). These data suggest that LANA 1–32 aa binding to histone could be due to highly charged residues of LANA 1–32 aa.

### Histones Bind to Amino as well as Carboxy Terminal Domains of LANA in-vitro

We further investigated in-vitro bindings of histones with larger domains of LANA by generating GST-fusion proteins of LANA-N (1–340 aa) and C-term (940–1162 aa) in E. coli. In-vitro translated histone H1 and H2B were incubated with GST-LANA-N and GST-LANA-C followed by washing the complex with wash buffer containing 300 mM of NaCl to increase the specificity. Detection of bound proteins showed that histone H1 primarily bound to LANA-N whereas H2B bound to both N- and C-terminal domains of LANA (relatively more to the C-terminus) (Fig. 5D). The binding of histones, H1 and H2B were unchanged when they were mixed together before binding to LANA GST proteins (Fig. 5F). Relative densities of H2B bound to C-terminus of LANA were slightly higher compared to the amino terminal domain of LANA (Fig. 5F). A representative coomassie stained image of GST LANA-N and LANA-C used in the binding assay is shown in figure 5F.

### GST Fused H1 and H2B Binds to LANA and CBD Alanine Mutants

To further support the binding data between histones and LANA we produced GST-fusion proteins of histone H1 and H2B in E. coli (Fig. 5G). LANA-FL with wt CBD and alanine substitution mutants (Fig. 5H) were in-vitro translated to determine direct binding with histones, H1 and H2B. Equal amounts of control GST, GST-H1 and GST-H2B were mixed with in-vitro translated products of LANA and its mutants. Detection of bound LANA after washing the complex with buffer containing 300 mM of NaCl showed binding of LANA with wt CBD and all alanine mutants to both the histones, H1 and H2B (Fig. 5I). These data suggest that H1 and H2B were able to bind to LANA directly and most likely to multiple domains of LANA.

We further detected the binding of LANA and its mutants expressed in 293T cells with GST-fused histones in order to determine whether post-translation modification of LANA can affect its binding affinity. Cells expressing Flag-tagged LANA and its mutants were lysed and equal amounts of lysates were incubated with GST, GST-H1 or GST-H2B. Detection of precipitated LANA after washing the complex with 300 mM of NaCl showed binding of LANA with both histones H1 as well H2B with almost similar affinity (Fig. 5J). All the CBD mutants (5–7, 8–10, 11–13, 14–15 and 5–15 aa) of LANA in full length construct showed similar binding to both histones (Fig. 5J). These data may suggest that histone H1 and H2B can bind to additional sites on LANA.

We further analyzed the binding of endogenous LANA from KSHV infected cells, BC3, BCBL1 and JSC1 with GST fusion proteins of histone H1 and H2B. Lysates from approximately 50 million cells were subjected to binding with control GST, GST-H1 or GST-H2B followed by washing the complex with RIPA buffer containing 300 mM NaCl to remove loosely bound proteins. Precipitated LANA detected by anti-LANA immunoblot showed comparable levels of LANA binding to histones, H1 and H2B GST fusion proteins (Fig. S5A). The lysates were also treated with DNase I to cleave the inter-nucleosomal DNA before binding with GST fusion proteins (Fig. S5B). Detection of LANA showed equivalent levels of LANA binding with both the histones (H1 and H2B) even with DNase I treatment. Lack of LANA signals with control GST confirms specificity of the binding assay (Fig. S5A and B). Uninfected BJAB cells did not show any LANA signal confirming specificity of LANA detection (Fig. S5A and B).

### LANA Colocalizes with Histones

**Figure 6:**
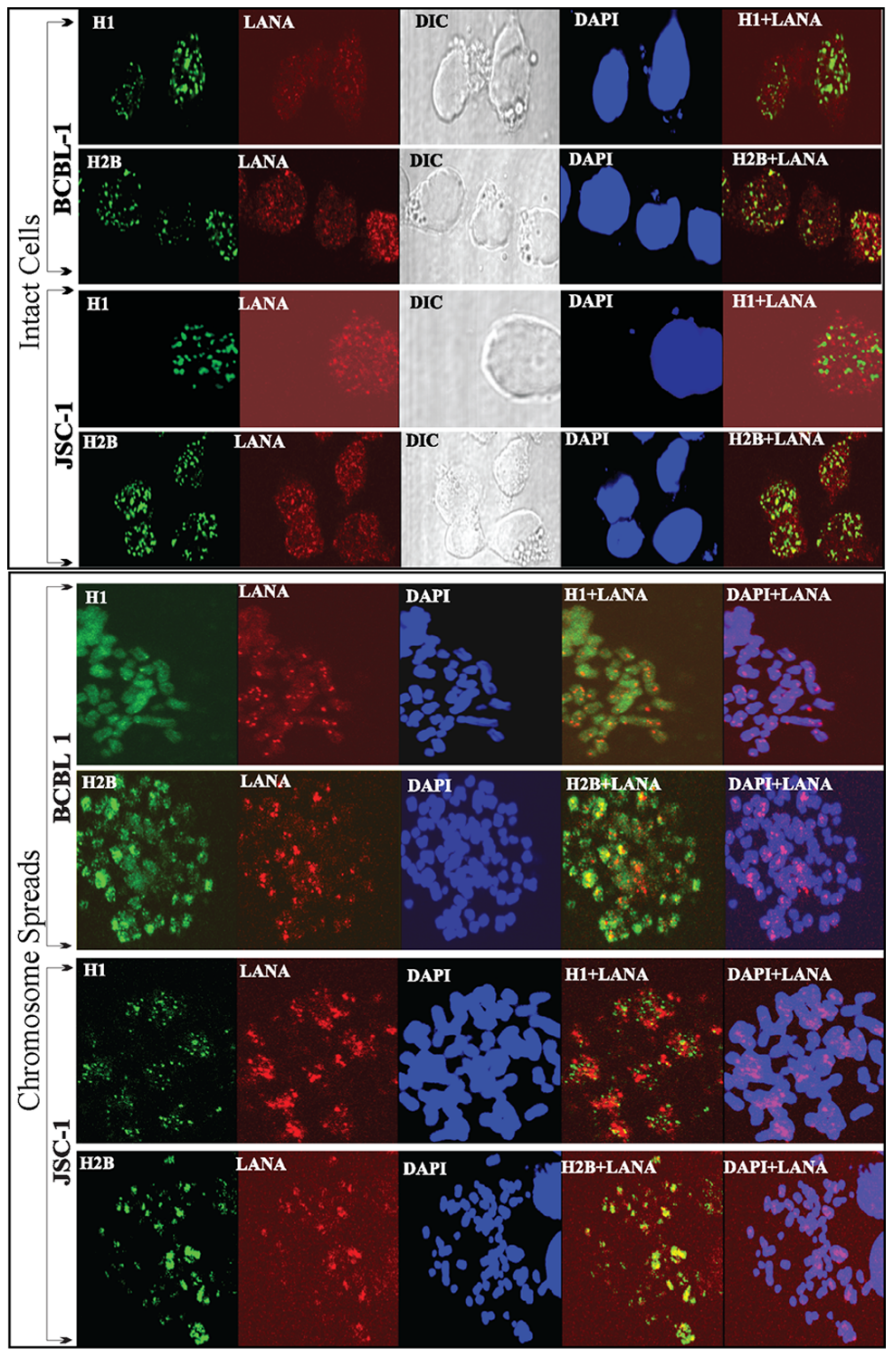
Immune localization of LANA and histones in PEL cells. In the ‘Intact Cells’ panel, KSHV infected, BCBL1 and JSC1 cells were fixed on a glass cover slip before permeabilization with TritonX-100. Histone H1 and H2B were detected with mouse anti-H1 and Rabbit anti-H2B antibodies. LANA was detected with rat anti-LANA monoclonal antibody. Histone H1 and H2B were detected with AlexaFluor 488 (green) and LANA was detected with AlexaFluor 594 (red). Nuclei were stained with DAPI shown in blue. Image including DIC were captured using Olympus confocal microscope. In the ‘Chromosome Spreads’ panel, BCBL1 and JSC1 cells were treated with hypotonic solution to prepare chromosome spreads. These spreads on glass slides were fixed and permeabilized with TritonX-100. Histone H1 and H2B were stained with specific antibodies and were counter stained with AlexaFluor 488 (green) and LANA were detected with AlexaFluor 594 (red). Yellow signals in the merge panels show colocalization.

Biochemical binding assays determined that both histones, H1 and H2B were capable of binding to LANA therefore, we determined whether these proteins were in close proximity during in-vivo conditions by immunolocalizing these proteins in intact cells as well as on chromosome spreads. Formaldehyde fixed BCBL1 and JSC1 cells were immunostained with anti-H1 and anti-H2B antibodies and co-stained with anti-LANA antibody. Detection of anti-histones with Alexa Fluor 488 (green) and anti-LANA with Alexa Fluor 594 (red) showed a large number of LANA signals colocalizing with histone H1 as well as H2B(Fig. 6, Intact Cells panel). Not surprisingly, LANA dots were also detected in the nucleus at spots without histone H1 or H2B signals possibly suggesting a complex mechanism through which LANA attaches to the chromosome. DAPI and DIC panels showed the nucleus and cell morphology, respectively.

In order to determine the localization of LANA on the chromosomes we prepared chromosome spreads of BCBL1 and JSC1 cells and localized histone H1 and H2B using specific antibodies. LANA signals were detected as punctate dots throughout the chromosome and importantly some of the signals overlapped with histone H1 as well as H2B yielding yellow signals (Fig. 6, Chromosome spreads panel). Histone H2B showed relatively higher levels of co-localization as compared to H1 on the chromosome spreads of both the cell lines, BCBL1 and JSC1 (Fig. 6, Compare the number of yellow dots in H1+LANA and H2B+LANA panels of chromosome spreads).

### PEL Cells Transduced with GFP-fused Histone H1 and H2B Showed Colocalization with LANA

**Figure 7:**
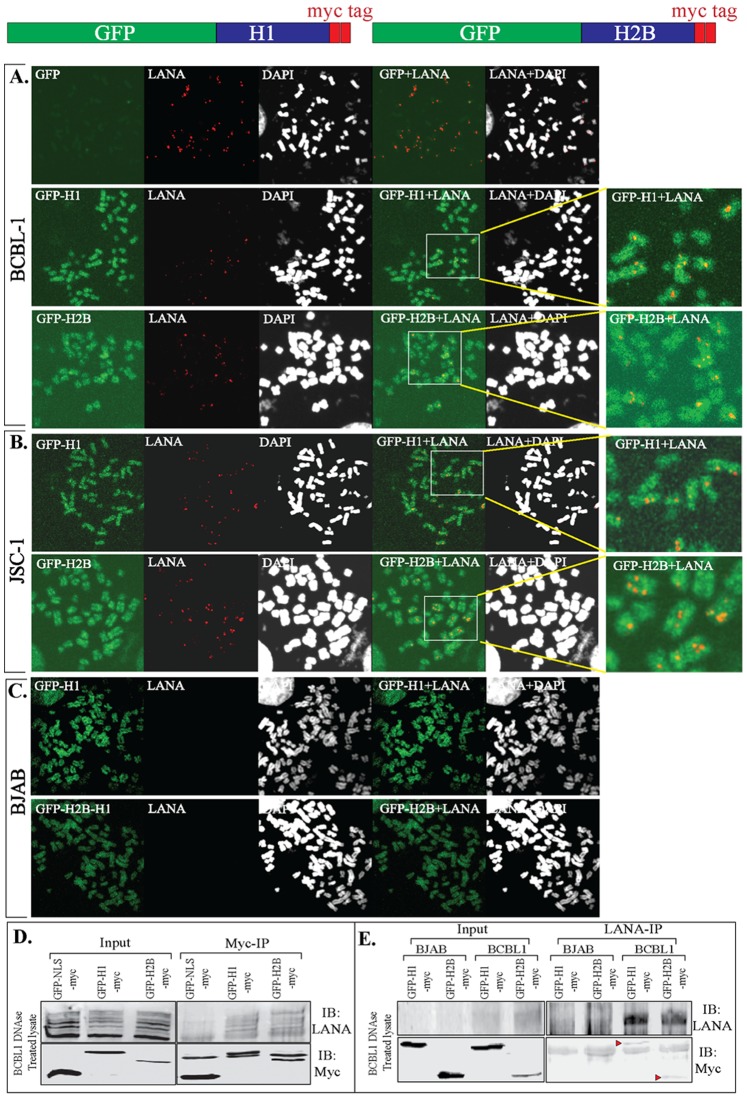
Localization of GFP-fused histone H1 and H2B with LANA in PEL cells. KSHV uninfected (BJAB) and infected (BCBL1 and JSC1) cells were transduced with lentivirus containing GFP-H1myc and GFP-H2Bmyc (schematic shown above panel A). Transduced cells were selected to obtain pure population of cells expressing GFP fused proteins. GFP-NLSmyc was used as control in this assay. **A.** Chromosome spreads of BCBL1 with GFP-H1 and GFP-H2B shows staining of entire chromosome with green but not in case of control GFP-NLSmyc. **B.** Chromosome spreads prepared from JSC1 cells stably expressing GFP-H1 and GFP-H2B. **C.** Chromosome spreads of BJAB cells expressing GFP-H1 and GFP-H2B. LANA were detected in above chromosome spreads using anti-LANA specific antibody. BCBL1 and JSC1 showed punctate dots throughout the chromosome, as expected. Lack of LANA signals in BJAB (KSHV negative cells) confirmed specificity of LANA detection. GFP-H1+LANA and GFP-H2B+LANA panels show yellow spots on the magnified view of the images. **D.** BCBL1 cells expressing GFP-NLSmyc, GFP-H1myc and GFP-H2Bmyc were lysed and the lysates were treated with DNase I before immunoprecipitation with anti-Myc antibody. Co-precipitating endogenous LANA was detected using anti-LANA antibody (IB:LANA). GFP and myc fused proteins were detected in anti-myc western blot (IB:myc). **E.** BJAB and BCBL1 cells stably expressing GFP-H1 or GFP-H2B were lysed and the lysates were treated with DNase I before immunoprecipitation with anti-LANA antibody (LANA-IP panels). Co-precipitating GFP-H1 and GFP-H2B were detected with anti-myc antibody (IB:myc).

In order to eliminate the potential bias in terms of the specificities of anti-histone H1 and H2B antibodies for the detection of respective signals, we used a uniform fluorescent (GFP) tag on both the histones and generated stable cell lines through lentivial vectors. GFP-H1 and GFP-H2B expressing cells, BCBL1, JSC1 and KSHV negative BJAB cells were subjected to preparation of chromosome spreads. Both histones, H1 and H2B fused to GFP uniformly stained the chromosomes of BCBL1, JSC1 and BJAB, as expected (Fig. 7A, B and C GFP panels). GFP-NLS-myc was unable to stain the chromosome of BCBL1 cells, used as control (Fig. 7A GFP). LANA signals were detected using anti-LANA antibody on BCBL1, JSC1 and BJAB cells. BCBL1 and JSC1 showed punctate staining pattern of LANA on the chromosome but not the BJAB cells thus confirming the specificity of LANA signals (Fig. 7, A, B and C, LANA panels). Merge panels of GFP and LANA showed co-localization or localization of LANA in close proximity to histone H1 as well as H2B (Fig. 7, A, B and C, GFP+LANA panels). Magnified view of histone H1 and H2B with LANA signals clearly showed comparable merge signals between H1 and H2B. Since these images were taken using confocal microscope on a single plane, yellow merge signals must be due to the close proximity of their localization. Chromosomes stained with DAPI are shown in white color and the LANA dots were localized to the chromosomes (Fig. 7, A, B and C, LANA+DAPI panels).

We also performed immunoprecipitation of GFP fused histones H1 and H2B with anti-myc antibody, since H1 and H2B were also fused with two-myc tags, to determine whether LANA can be co-immunoprecipitated along with histones. GFP-NLS-myc was used as control in this assay. Lysates were treated with DNase I before immunoprecipitation with anti-myc antibody. Comparable levels of LANA co-immunoprecipitating with both H1 and H2B were detected (Fig. 7D). Expressions of GFP-NLS-myc (control), GFP-H1-myc and GFP-H2B-myc in the input and Myc-IP panels were detected with anti-myc blot (Fig. 7D). We also performed reverse IP with anti-LANA antibody to detect co-precipitating GFP fused H1 and H2B with anti-myc antibody. We used BJAB with GFP-H1- and H2B-myc for anti-LANA IP as control. The lysates were treated with DNase I before anti-LANA antibody immunoprecipitation and immune detection of co-precipitating GFP-H1 and GFP-H2B in WB. We detected co-precipitations of GFP-H1-myc and GFP-H2B-myc in BCBL1 cells but not in LANA lacking BJAB suggesting specific association of both histones with LANA (Fig. 7E).

**Figure 8:**
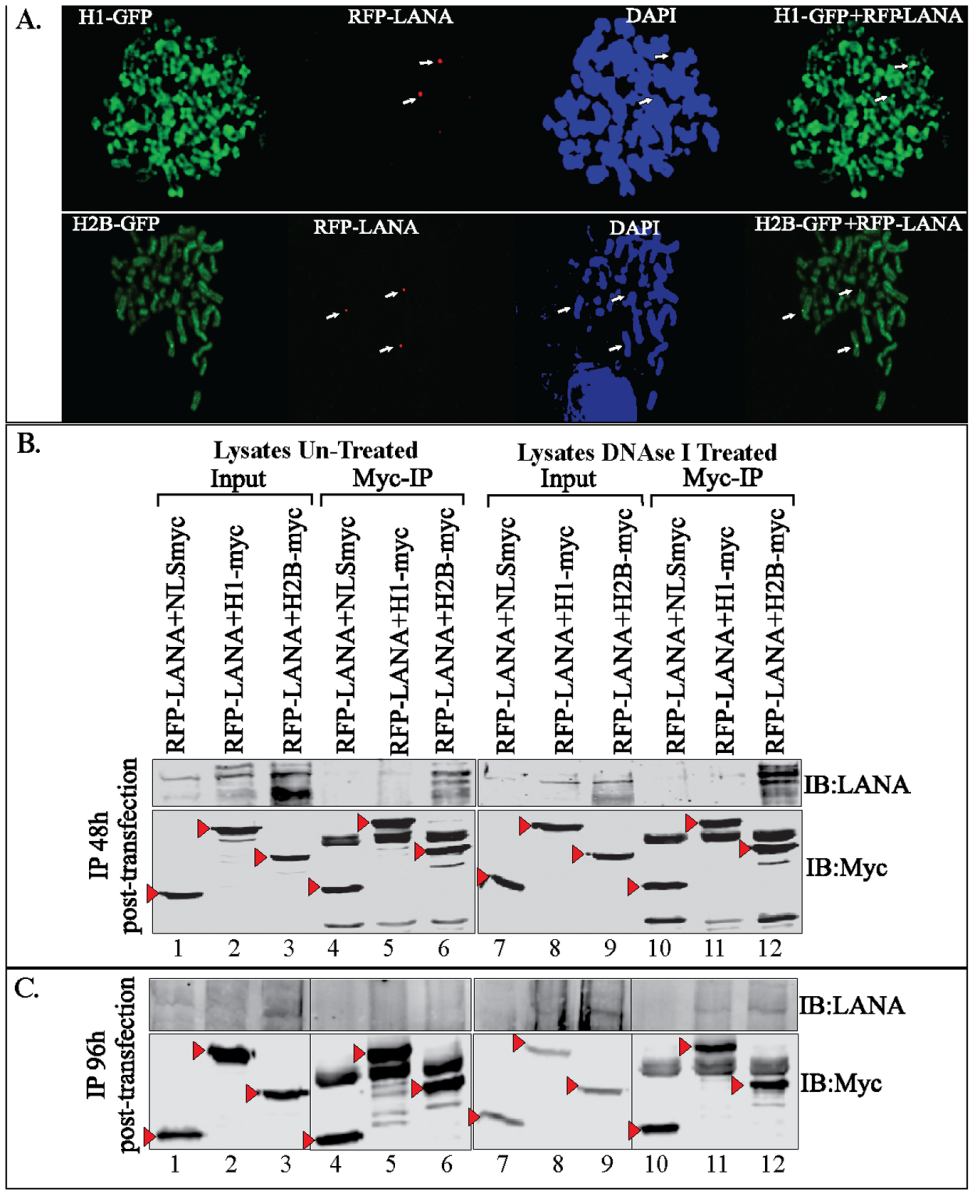
RFP-LANA associated with GFP-fused histone H1 and H2B. **A.** Chromosome spreads of 293T cells stably expressing GFP-H1 and GFP-H2B with RFP-LANA. GFP-H1 and GFP-H2B uniformly stained the entire chromosome and RFP-LANA showed distinct punctate localization on the chromosome detected by DAPI staining. **B.** 293T cells transfected with RFP-LANA and NLS-myc (lane 1), GFP-H1myc (lane 2) and GFP-H2Bmyc (lane 3) were harvested after 48 h post-transfection and lysed in RIPA buffer for immunoprecipitation with anti-myc antibody. Lysates from the above-mentioned transfection were treated with DNase I in second set before anti-myc immunoprecipitation. Bright band of RFP-LANA was detected in Myc-IP panels with GFP-H2Bmyc in untreated (lane 6) as well as DNase I treated panels (lane 12). GFP-NLS-myc, GFP-H1myc and GFP-H2Bmyc in the input and IP lanes were detected with anti-myc WB and are indicated with red triangle. **C**. 10% of the above-transfected cells were passaged and allowed to grow for 96 h before lysing them for anti-myc immunoprecipitation. Lysates were either untreated or DNase I treated before anti-myc immuneprecipitation. Co-precipitating RFP-LANA was detected using anti-LANA western blot (IB:LANA). GFP-NLS-myc, GFP-H1myc and GFP-H2Bmyc in the inputs and IP lanes were detected with anti-myc WB (IB:myc).

To determine whether the amounts of LANA present may affect it’s binding to histones, we performed time course immunoprecipitation of histones fused with GFP and detected co-precipitation of RFP-LANA. Chromosome spreads of cells stably maintaining GFP-H1 or GFP-H2B with LANA RFP showed chromosome painting by the GFP-fused histones and punctate dots of RFP-LANA on the chromosome (Fig. 8A). Immunoprecipitation of GFP-H1-myc and GFP-H2B-myc co-expressing LANA at 48 h post-transfection, when the expression of LANA was very abundant, detected LANA primarily with H2B (Fig. 8B, a faint signal in H1 lane compared to H2B lane). RFP-LANA with control GFP-NLS-myc did not show LANA in the immunoprecipitated lanes suggesting specific co-precipitation of LANA with histones (Fig. 8B). Treating the lysates with DNase I to eliminate inter-nucleosomal DNA showed similar results (Fig. 8B, lysates DNase I treated panel). Interestingly, immunoprecipitation of histones after the first passage (10% of the cells from 48 h post-transfection were seeded) at 96 h post-transfection, when the expression of LANA was subtle, both GFP-H1 and GFP-H2B were able to precipitate low amounts of LANA (Fig. 8C). Additionally, treatment of lysates with DNase I showed similar enhanced precipitation of RFP-LANA in both GFP-H1 and GFP-H2B lanes (Fig. 8C). Control lanes, RFP-LANA with GFP-NLS-myc did not now LANA immunoprecipitation, as expected (Fig. 8C). These results suggest that during expression when the LANA amounts are significantly higher it may associate with histones, which are highly acidic proteins. However, when the expression of LANA reaches levels similar to KSHV infected cells, it may have appropriate physiological binding to both histone proteins being investigated.

### Split GFP Complementation Assay Showed High Affinity of LANA 1–32 to Histone H2B as Compared to Full Length LANA

**Figure 9:**
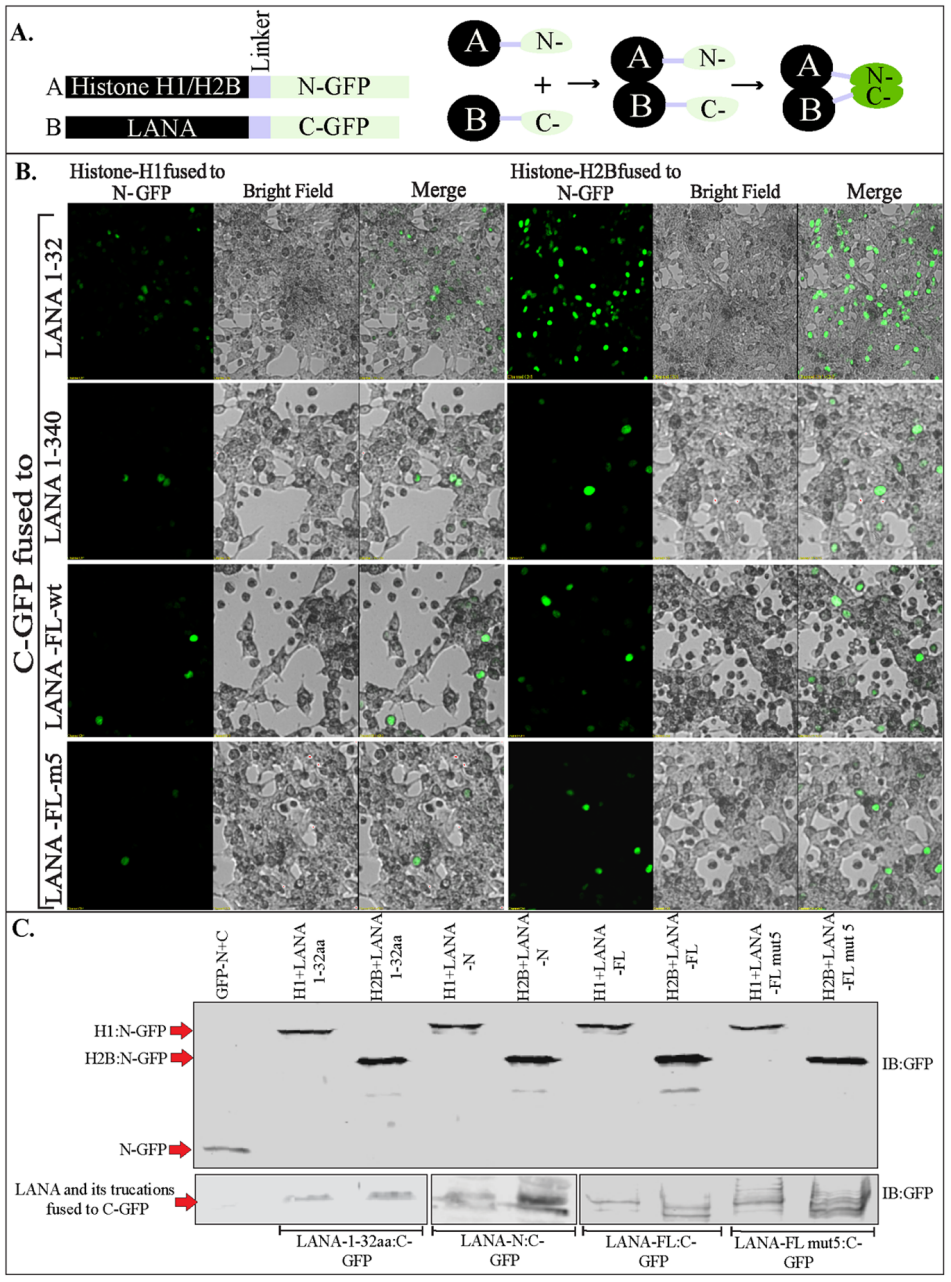
Split GFP complementation assay to determine in-vivo association of LANA with histones. **A.** Schematic of split GFP fusion proteins and their association. **B.** Histone H1 or H2B fused with GFP-N term (1–157 aa) were co-transfected with LANA1–32 aa (top panel), LANA 1–340 aa (second panel), LANA-FL with wt CBD (third panel from top) and LANA-FL with alanine substituted 5–15 aa of CBD (bottom panel) fused with C-term of GFP (158–238 aa). GFP fluorescence was imaged after 48 h post- transfection. **C.** Western blot with anti-GFP antibody to show relatively similar expression of GFP-N term and C-term fused proteins in cells used for comparison of fluorescence. Band of interests are indicated with red arrow.

Protein-fragment complementation assay (PCA), which employs fluorescence reporters such as green fluorescent protein (GFP) also referred to as Bimolecular Fluorescent Complementation (BiFC), is a recent addition to the biochemical methods for determining protein-protein interactions in-vivo. The advantage of BiFC is that it does not require immune localization of proteins and therefore can rapidly determine the specific cellular locations of proteins without having to worry about background signals [42]–[44]. In this assay, the intact GFP proteins are separated into two spontaneously associating fragments to form the GFP fluorophore when the GFP fragments are brought together through the interacting proteins [45], [46]. To determine the split GFP complementation and obtain information of LANA’s interaction with these two histones, we transfected histone H1 and H2B fused with N-terminus of GFP along with different truncation mutants of LANA fused to the C-terminus of GFP (Fig. 9A). Green fluorescence captured at the same settings on the microscope showed significantly higher fluorescence of LANA 1–32 aa transfected with H2B as compared to H1 (Fig. 9B 1–32 aa panels) suggesting that LANA 1–32 aa has higher affinity. However, when LANA1–340 aa fused to GFP-C terminus was transfected with histones the difference in fluorescence between histone H1 and H2B was not as evident as that seen with LANA 1–32 aa. These experiments were performed multiple times and a representative image is presented in figure 9. The fluorescence with LANA-FL fused with GFP-C terminus was almost similar to LANA 1–340 aa (Fig. 9B, LANA-FL-wt panel). We also detected fluorescence with both the histone, H1 and H2B when co-transfected with LANA-FL containing CBD mutated to alanine, mut 5 (Fig. 9B, LANA-FL-mt5), suggesting that histone H1 and H2B were able to bind to other regions of LANA. Arguably, the fluorescence from H2B co-transfected with LANA was higher as compared to histone H1 but LANA was also able to compartmentalize with histone H1. For the record, split GFP fragments, GFP-N and -C termini were unable to associate and yield green fluorescence. Strikingly, the association of LANA 1–32 aa with H2B was the strongest suggesting that conformation of LANA 1–32 aa might allow efficient interaction with histone H2B, but this may not entirely reflect the conformation of the full length LANA molecule. The expression levels of GFP-N terminus fused histone H1 and H2B were comparable, which were detected by anti-GFP antibody (Fig. 9C). GFP-C terminus fused with LANA 1–32 aa, 1–340 aa and LANA-FL were also comparable between cells transfected with histone H1 and H2B (Fig. 9C).

Since LANA 1–32 aa had strong affinity to histone H2B, we determined whether mutating CBD of 1–32 aa had any affect on in-vivo association by transfecting equal amounts of wt LANA 1–32 and CBD mutated (5–15 aa to alanine) LANA 1–32 with H2B fused to the GFP-N terminus. Fluorescence detected under same conditions showed drastic decrease in fluorescence when the CBD was mutated (Fig. S6A) with the same levels of proteins expression detected by GFP WB (Fig. S6B). This confirmed previous observation that LANA 1–32 aa strongly binds to histone H2B, and a mutation of CBD depletes their association [20]. Histone H1 fused to GFP-N terminus transfected with LANA 1–32 aa showed detectable levels of fluorescence as detected in an earlier experiment, which was also abolished in CBD mutant of LANA (Fig. S6A). Control transfections were done each time and representative images of the controls are shown in Figure S6C. GFP-N terminus were transfected with GFP-C term, GFP-LANA 1–32 aa, 1–32 mut5, GFP LANA-FL and mut 5 and none of them showed any green fluorescence despite similar protein expression detected by Western blot (Fig. S6 C and D). Similarly, GFP-C terminus co-transfected with GFP-N terminus fused histone H1 and H2B did not show any green fluorescence under similar conditions (Fig. S6C). GFP-N fused with H1 and H2B were detected in Western blot. GFP-C terminus by itself was not detected due to a very small size of the protein (amino acid 158–238 aa) in this gel system.

### Fluorescence Resonance Energy Transfer (FRET) Revealed Association of LANA with Histone H1 and H2B

Immune localization assays determine the colocalization of proteins but the proximities of their localization in living cells is accurately determined by a transfer of resonance energy in a fluorescence resonance energy transfer (FRET) assay [47]–[49]. The FRET assay is based on a compatible GFP pair to be used as a donor and acceptor. The Cyan Fluorescent Proteins (CFP) and Yellow Fluorescent Proteins (YFP) are a commonly used fluorescent protein pair in a sensitized emissions assay [50]. Consideration for selection of a good FRET pair is that the donor emission should have maximum overlap with the excitation spectra of acceptor protein [50]. The excitation wavelength for ECFP is 408 nm and the emission is at 477 nm, which falls close to the excitation wavelength of YFP (514 nm). FRET index is a relative way of presenting the FRET efficiency, which varies with changes in energy transfer associated with donor-acceptor configuration. FRET index increases and decreases upon an increase or decrease in FRET efficiency, respectively. FRET indices are useful for qualitative studies and also to determine the relative measurements within the same experiment.

**Figure 10:**
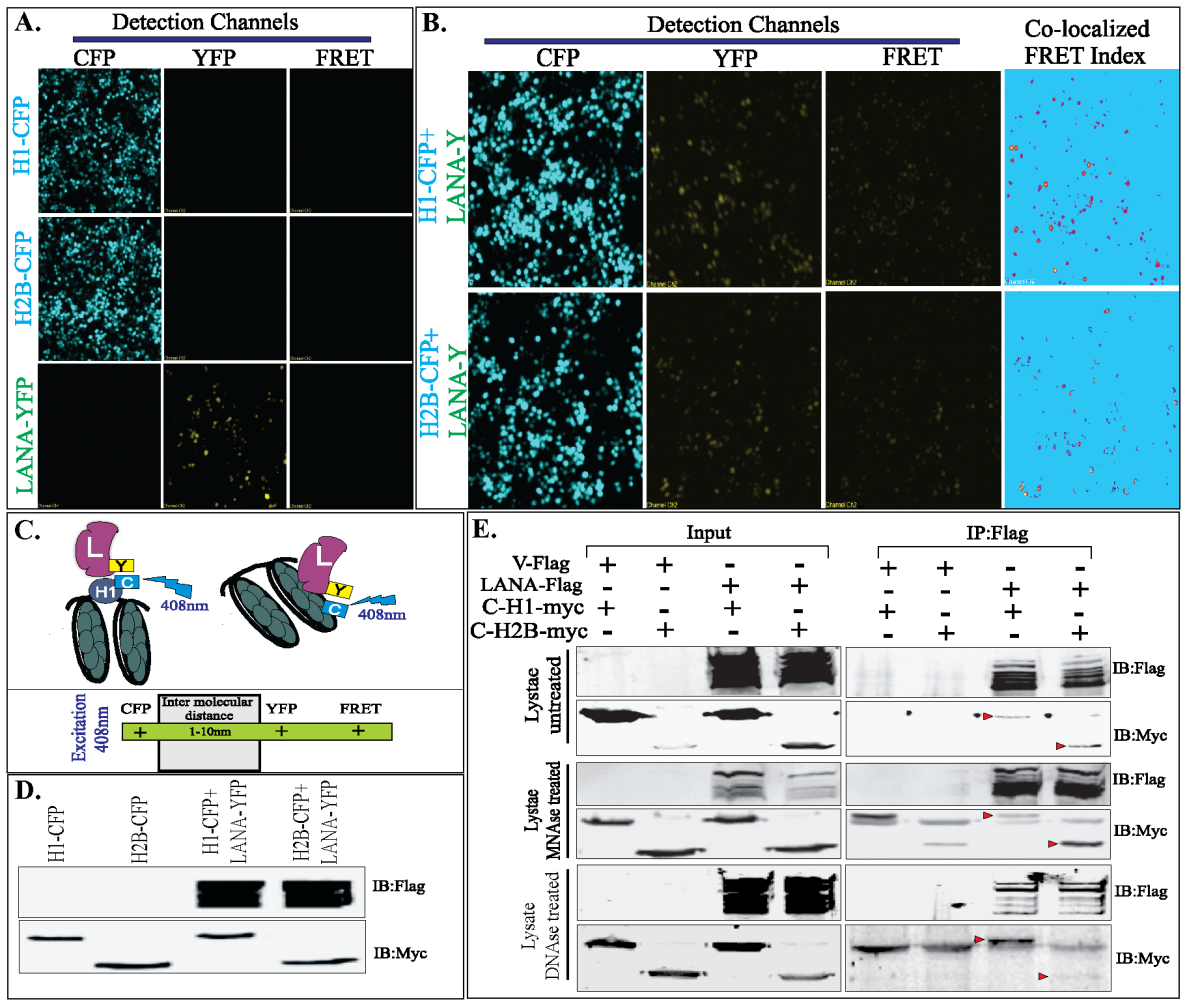
Analysis of Fluorescence Resonance Energy Transfer (FRET) between LANA and histones. **A.** 293T cells were stably transduced with CFP-fused H1myc or CFP-fused H2Bmyc. YFP-LANA-Flag was transfected alone in H1-CFP or H2B-CFP expressing cells. To capture the control images for FRET analysis, H1-CFP and H2B-CFP cells were excited with 405 nm laser to detect any bleed through in YFP emission and also signals in FRET channel. Similarly, YFP-LANA was excited with 515 mm to detect the fluorescence in YFP channel as we all FRET Channel. These individually expressing proteins did not show any signals in FRET channel. **B.** H1-CFP+ LANA-YFP and H2B-CFP+LANA-YFP expressing cells were excited with 405 nm laser to excite CFP proteins, which emits at 477 nm (cyan). Emission spectra of CFP fall in the excitation range of YFP (514 nm). Therefore, based on the proximity of the proteins to transfer energy from the donor to acceptor, emissions from donor can excite the acceptor and thus there is FRET. Emission from CFP fused with H1 and H2B were able to excite YFP-fused with LANA to emit yellow (535 nm) signals. FRET Channel detected almost similar levels of signals in both H1 and H2B transfected with LANA. Co-localized FRET index or FRET efficiency were calculated ImageJ software, which showed comparable localization of H1 and H2B with LANA. **C.** Proposed model of LANA’s interaction with histone H1 and H2B fused with CFP. For an efficient FRET the intermolecular distance is deciding factor and the molecules separated by less than 10 nm can transfer energy to yield FRET signals. **D.** Western blot with anti-myc to show the expressions of CFP-fused H1 and H2B and anti-Flag to show comparable expression of YFP-LANA-Flag in those cells. **E:** Cells used in FRET assay show binding of LANA with histone H1 and H2B after treatment with DNase I as well as MNase. CFP-H1-myc, and CFP-H2B-myc stably expressing in 293T cells were transfected with Flag vector or YFP-LANA-Flag. Lysates from these cells were divided into three parts, i) untreated, ii) MNase treated, iii) DNase I treated followed by immunoprecipitation of LANA with anti-Flag antibody. Immunoprecipitated LANA in all the set was detected with anti-Flag blot (IB:Flag). Co-precipitating H1 and H2B fused to CFP were detected with anti-myc blot (IB:myc). Detection of H1 and H2B in all three conditions (untreated, MNase and DNase I treated) in LANA expressing cells, but not in vector transfected cells, confirmed specific association of both histones with LANA.

Similar to any fluorescence measurement, calculation of the FRET index needs to account for 1), bleed-through in excitation, i.e., when the acceptor is excited by the donor's excitation wavelength and vice versa; and 2), cross talk in emission detection, i.e., when the emission of a donor contributes to the signal measured in acceptor detection channel, and vice versa. We normalized the bleed through by reducing the laser intensities to have minimal emission in YFP channel when excited CFP-fused histones (Fig 10.A). Similarly, emission of YFP-LANA was detected by exciting the YFP (Fig. 10A). These images were used for the calculation of FRET indices using ImageJ software and algorithms [39], [51]. Cells expressing both proteins, CFP fused histone H1 with YFP-fused LANA-FL and CFP fused H2B with YFP-fused LANA-FL (Fig. 10B). Excitation of CFP in both samples lead to emission of YFP channel when both proteins were present (Fig. 10B). Co-localized FRET-indices were calculated by importing these images to ImageJ software and the purple signal indicated the co-localized signals (Fig. 10B, Co-localized FRET channel). Importantly, the levels of colocalized signals were comparable between cells expressing histone H1 or H2B with LANA (Fig. 10B, Co-localized FRET channel). For efficient energy transfer between the donor and acceptor, inter molecular distance plays a critical role and the molecules located within a 10 nm distance can transfer energy to an appropriate acceptor (Fig. 10C) [51], [52]. Expression of CFP-fused histones H1 and H2B and YFP-fused LANA were detected by anti-myc (histones) and anti-Flag (LANA) in the cells used in FRET assay, which showed comparable expression between samples (Fig. 10D).

Additionally, we performed imunoprecipitation on the cells used in FRET, which were stably expressing histones, H1 and H2B fused to CFP-myc. 293T cells expressing CFP-H1-myc and CFP-H2B-myc were transfected with either flag vector and LANA-Flag and the lysates were divided into three parts, i) untreated, ii) MNase treated and iii) DNase I treated. Immunoprecipitation of LANA with anti-Flag antibody co-precipitated both the histones, CFP-fused H1 and H2B in all three above-mentioned conditions detected by anti-myc WB (Fig. 10E). Specificity of LANA association was confirmed as vector transfected cells did not show any co-precipitating histones (Fig. 10E). These data confirms association of histones independent of intercalating DNA at least in our expression systems.

In a reverse immunoprecipitation experiments from cells stably expressing YFP-fused LANA-Flag and NLS-myc, CFP-H1myc or CFP-H2Bmyc were subjected for anti-myc IP after treating the lysates with DNase I. Immune detection of YFP-LANA by anti-Flag antibody showed LANA being co-precipitated with both CFP-H1 as well as CFP-H2B but not with GFP-NLSmyc (Fig. S7). GFP-NLSmyc, CFP-H1myc and CFP-H2Bmyc in the input and IP panels were detected with anti-myc WB (Fig. S7). These results again support an association of LANA and the histones, H1 and H2B.

### Different Variants of Histone H1 was Capable of Binding to LANA

**Figure 11:**
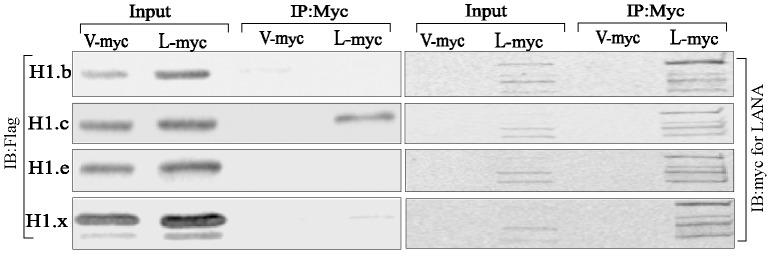
Histone H1 variants associated with LANA. H1 variants were cloned into a Flag epitope vector and transfected with a vector containing LANA-myc. Histone H2B-Flag was also transfected for comparison. Anti-myc IP to precipitate LANA co-precipitated H1.c as well as H1.x, the binding affinity of H1.c relatively stronger then H1.x. LANA in the input and IP lanes were detected in anti-myc blot.

Throughout the over-expression system, we used H1.x variants of histone H1 however, there are other variants of H1 in human cells but the clones expressing those genes were not available until recently. These variants are H1.1 to H1.5, H1.0, and H1x, which correspond to the coding genes, H1.a (H1.1), H1.c (H1.2), H1.d (H1.3), H1.e (H1.4), H1.b (H1.5), H1.F0 (H1.0) and H1.x (H1.Fx) [53]. Among these, H1.c, H1.d, H1.e H1.b and H1x are ubiquitously expressed, H1.a is restricted to certain tissues, and H1.F0 accumulates in terminally differentiated cells [53]. Therefore, we acquired the coding sequences of H1.b, H1.c and H1.e to test their binding with LANA. Expression of Flag-tagged histones with myc vector of LANA-myc and immunoprecipitation with anti-myc antibody showed efficient co-precipitation of H1.c (Fig. 11). Comparison of co-precipitated H1.c with H1.x showed that LANA is able to bind even more strongly to H1.c (Fig. 11). Expressions of LANA in input as well as IP lanes were detected with anti-myc Western blot. These data suggest that LANA is capable of binding to multiple variants of linker histones.

### LANA Binds with the Histones H1, H2B as well as the High Mobility Group (HMG) Proteins

**Figure 12:**
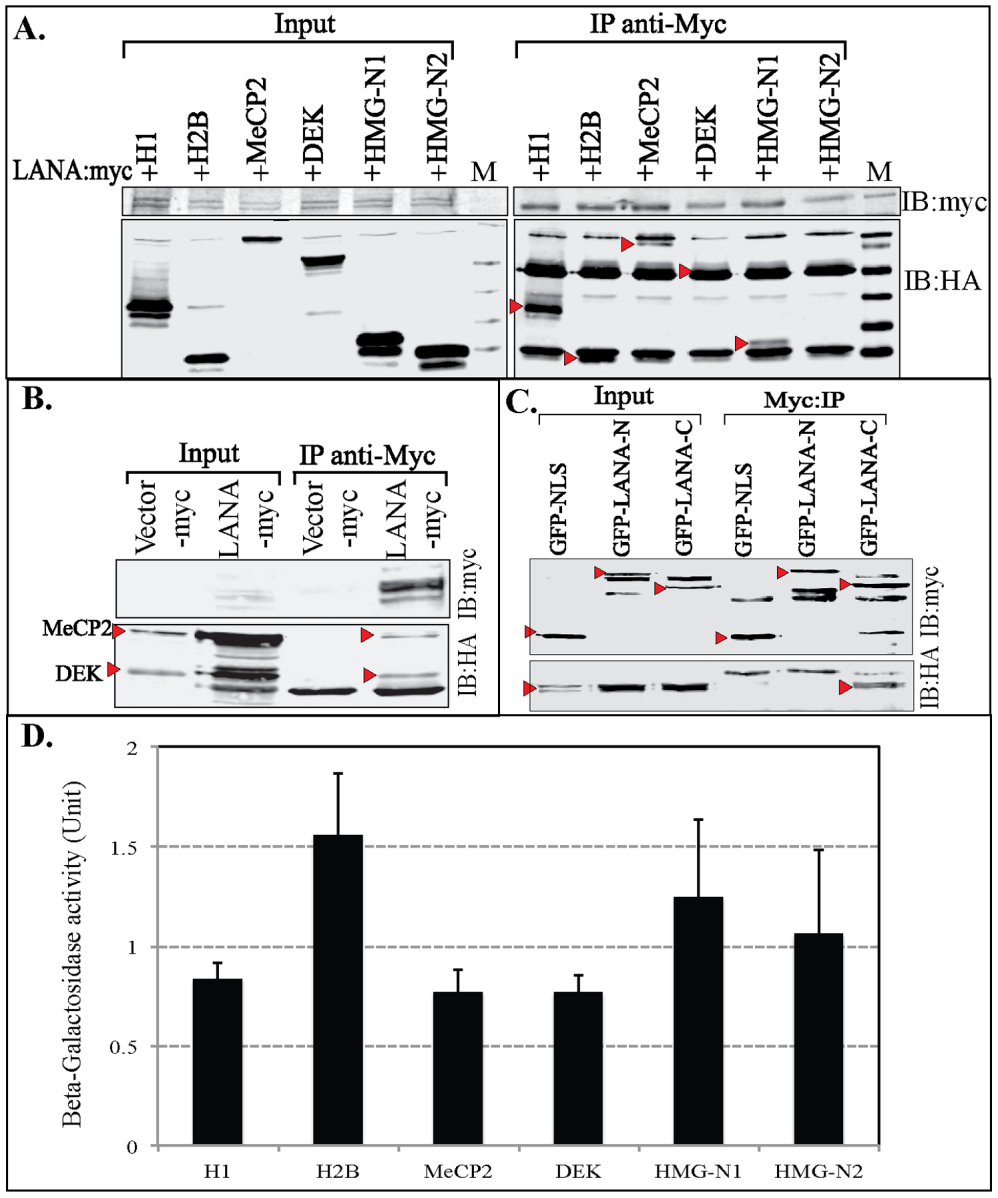
LANA associates with histones and non-histones nucleosomal proteins. **A.** HA tagged Histone H1, H2B, MeCP2, DEK, HMG-N1 and HMG-N2 were co-transfected with LANA-myc. LANA was precipitated with anti-myc antibody and co-precipitating proteins were detected with anti-HA blot (IB:HA). M is the marker lane. Co-immunoprecipitating proteins are indicated with red triangle. **B.** HA tagged MeCP2 and DEK together were transfected with myc vector or LANA-myc followed by anti-myc immunoprecipitation (IP). The complex were resolved to separate DEK from the heavy chain and immune detection with anti-HA confirmed binding of both MeCP2 and DEK to LANA. **C.** HA tagged MeCP2 was transfected with LANA-N or LANA-C (fused to GFP and myc) along with control GFP-NLS-myc followed by anti-myc immunoprecipitation. Detection of MeCP2 with anti-HA blot showed its binding with C-terms of LANA (IB:HA, red triangle). GFP-NLS-myc, GFP-LANA-N and GFP-LANA-C in the input and IP lanes are indicated by red triangle. **D.** Beta-galactosidase activity of LANA interacting proteins. Yeast 190 stable expressing LANA-N term fused to GAL4DBD was co-transformed with LANA interacting proteins (H1, H2B, MeCP2, DEK, HMG-N1 and HMG-N2) fused to ACT domain of GAL4. Induction of beta-galactosidase due to the interaction of LANA bait with prey was detected by ONPG assay. Relative activity normalized against LANA-N fused to GAL4DBD with vector GAL4ACT, are shown in the bar graph from three independent experiments with three independent clones. Standard error bars were calculated based on the data from three independent experiments. Luciferase fused GAL4ACT was used as a negative control, which showed no induction of beta-galactosidase activity.

We further compared the binding of known LANA interacting proteins, MeCP2 and DEK with histones, H1, H2B and non-histone nucleosomal proteins, HMG-N1 and HMG-N2. HMGs are abundantly expressed nuclear proteins, which lack DNA binding sequence but can bind to cellular chromatin and can alter the complexity of chromatin structure [54], [55]. Human cells have four groups of HMGN proteins, which include HMGN1 (HMG14), HMGN2 (HMG17), HMGN3 (Trip7) and HMGN4 [56]. Between these, HMGN1 and HMGN2 are the most abundantly expressed and estimated to be more than one molecule per nucleosome [57], [58]. HMGN proteins bind to the chromatins of transcriptionally active genes and are considered to be important for generation and maintenance of open chromatin [59]. Since LANA binds to both open and condensed chromatin, we wanted to determine whether LANA is capable of binding to non-histone nucleosomal proteins. Co-transfection of histone H1, H2B, MeCP2, DEK, HMGN1 and HMGN2 with LANA showed precipitation of histone H1, H2B, MeCP2, DEK and HMGN1, indicated by red triangle (Fig. 12A, IB:HA). Expressions of these proteins with myc vector did not precipitate these proteins (data not shown), which show specificity of their association with LANA. Binding of HMGN2 could not be convincingly detected because it bands with light chain (Fig. 12A, HMGN2 lane). Binding of DEK and MeCP2 were confirmed by co-expressing them together along with LANA or vector control. Immunoprecipitation of LANA showed binding with both DEK and MeCP2 as reported earlier (Fig. 12B) [19]. Binding of MeCP2 to LANA domains, by co-immunoprecipitation identified MeCP2 binding in the C-terminal domain of LANA, as reported recently (Fig. 12C) [24].

We further determined relative interaction of these proteins by performing yeast-2-hybrid assay in which interaction was measured by the induction of beta-galactosidase activity [19], [60]. Yeast cells stably expressing Gal4DBD-LANA were co-transformed with these proteins fused with Gal4ACT and empty vector as negative control followed by measuring the induction of beta-galactosidase activity. The relative beta-galactosidase activities were normalized against empty vector (Fig. 12D). The data from three independent experiments showed that histone H2B had strongest association but that the other known and newly tested molecules also induced beta-galactosidase activity (Fig. 12D). These data suggests that LANA can associate with various nucleosomal proteins and the interaction may depend on their association and physiological state of the cells.

## Discussion

KSHV, like other herpesvirus establishes a life long latent infection and can induce tumoriegenesis similar to the other members of the gammaherpesvirus family [22], [41], [61]. Induction of tumorigenesis results into faster cells growth and cell division. KSHV genome replicates along with cellular DNA by using the host-cellular replication machinery [62]. Replicated viral genome segregates into the dividing tumor cells with the help of LANA protein, which binds to KSHV genome as well as the host chromatin throughout cell cycle [20], [21], [32], [63]. LANA achieves this function by binding to various nucleosomal proteins including histones, nuclear mitotic apparatus (NuMA), centromeric protein F (CENP-F) and a kinetochore protein, Bub1 [21], [32]. Depletion of these proteins from KSHV infected cells have shown inhibitory effects on the ability of the KSHV genome to passage to the nascent daughter cells after cell division, thus supporting their role in segregation [21], [32]. LANA has been shown to dock on the surface of core histone H2A and H2B of the nucleosomes [20], [63]. However, LANA is also capable of binding to proteins of heterochromatin region including heterochromatin protein 1 (HP1) and methyl-CpG-binding protein 2 (MeCP2), which are implicated in transcriptional silencing of genes in CpG-methylated regions as well as the activation of euchromatic genes [19], [23], [24]. LANA accumulates in the heterochromatin region as pericentric punctate dots, which is proposed to be due to its binding with MeCP2 [24], [64]. Although LANA can bind to H2B/H2A of the nucleosomes, its attachment on the heterochromatin region, which has compact chromatin with H2B/H2A surface unavailable for binding to a large protein such as LANA, suggests it’s ability to bind with additional nucleosomal proteins. Histone H1 is required for compaction of the nucleosome [65], [66], and was previously shown to bind with LANA. Another protein that binds to heterochromatin region is MeCP2 and was recently confirmed to be important for targeting LANA to the heterochromatin [24]. This led us to more comprehensively determine the binding of LANA with histone and non-histone proteins of the nucleosome using various biochemical assays.

Binding of histone H1 and H2B from purified histones with in-vitro translated LANA confirmed the binding of both H1 and H2B histones. Histone H2B antibody showed a very distinct band as compared to histone H1, probably due to difference in the specificities of these antibody that may have contributed to the lack of histone H1 detection in the previous study [20]. A bright band of H2B was further detected from the lysates of BCBL1 cells, which specifically co-precipitated LANA as compared to a faint band of histone H1. However, when we compared the levels of histone H1 and H2B in the input lanes, the band intensities of these proteins were very different confirming the differences in the specificity of these antibodies. Regardless of the band intensities, both histones H1 and H2B were able to bind with LANA from the lysate similar to purified histones. Stable expression of LANA 1–32 aa in BJAB precipitated H2B but not a detectable level of H1 suggesting involvement of additional domains of LANA for binding to H1. Binding in expression systems showed a very faint signal of histone H1 with 1–32 domain of LANA with wt CBD as compared to histone H2B again confirmed that LANA 1–32 aa binds very strongly to histone H2B. Introduction of alanines to replace various residues of CBD in LANA 1–32 aa showed that residue 5–13 aa are critical for H2B binding as determined in the precious study [20]. Treatment of the lysates with DNase I was unable to eliminate the binding of LANA 1–32 aa binding to H2B thus confirmed that residues 5–13 aa of LANA directly interacts with acidic patch formed by H2A-H2B within the nucleosome determined by the x-ray crystallography [20]. A small peptide of LANA 1–23 aa could easily get access to the surface of core histones, even on a compact chromatin, but whether full length LANA can access the nucleosome surface for docking on the chromosome is not fully understood. Although increasing the length of LANA from 1–32 aa to amino terminus (1–340 aa) and to the full length in the immunoprecipitation assays specifically precipitated histone H2B, which shows that LANA can bind to H2B present on free nucleosomes of the lysed cells, it does not address whether LANA can access the surface of H2A-H2B of condensed heterochromatins.

Detection of histone H1, although lower than H2B in immunoprecipitations with LANA 1–340 aa and full length, even with DNase I treatment, suggest some levels of LANA association with H1 as well as other nucleosomal proteins. We presume that LANA binds to histone H2B on open chromatin during DNA synthesis and mitotic phase of the cell cycle. Data of previous study on histone H2B immunoprecipitation with LANA 1–32 aa from mitotic and non-mitotic cells could be due to the fact that i) a small region of LANA may get access to the surface of nucleosomes of the non-mitotic cells, ii) lysis of the cells generates mono-nucleosomes and thus the surface become available for binding to LANA. Treatment of the cell lysates with DNase I and micrococcal nuclease cleaved the intercalating DNA, which may assist in hitching indirect interactions to directly interacting proteins. However, at the same time treatment with DNase I can eliminate the DNA wrapped around the nucleosome, which disrupts nucleosome assembly and “frees up” the histones. Similarly, treatment with MNase cleaves the DNA in between the nucleosomes to generate mono-nucleosomes [67]. Treatment of MNase also removes the complexity of the host chromatin and thus may not reflect a direct binding of LANA to the surface of compact chromatin. Therefore an assay, which allows detection of LANA on the chromosome in native state, would be an ideal to determine the exact mechanism of LANA’s binding to the host nucleosome.

All the biochemical assays including in-vitro bindings of purified GST fusion proteins of LANA-N with in-vitro translated histone H1 and H2B efficiently bound to both the histones, H2B as well as H1. The results of these proteins synthesized in eukaryotic cells, to have appropriate post-translational modification, were no different than the results of in-vitro translated proteins. Additionally, purified histones fused to GST and synthesized in *E. coli* bound with full length LANA translated in-vitro as well as expressed in eukaryotic cells, showed binding with both the histones, H1 and H2B. These data conclusively confirmed that LANA has the ability to bind with purified histones.

In an attempt to determine the association of LANA with nucleosomal proteins by immunolocalization assay using specific antibodies and confocal microscopy, we found expected punctate localization of LANA, which overlapped with histone H1 as well as histone H2B signals. The localization of the histone confirmed that both the histones are in close proximity to LANA, however, one may argue that all the histone proteins will colocalize, if any of the histone colocalize as they all are part of the nucleosome and thus they remain in close proximity. Importantly, the pattern of LANA colocalization with histone H2B was slightly different than H1 with H2B having more colocalized spots in both the cell lines suggesting specificity in their interaction with LANA. Localization of these proteins on chromosome spreads also showed similar results of LANA being colocalized with H2B at a relatively greater number of spots than histone H1. However, this again came down to whether the antibody specificities of both proteins were similar. We tackled this by using a lentiviral system to express fluorescently fused histone H1 and H2B into KSHV infected, BCBL1 and JSC1 cells. This ensured that both the histones were expressed and localized at physiological states by the fluorescent tag (GFP) not by the antibody. Chromosome spreads of the cells with stable GFP-H1 and H2B showed uniformed staining of the chromosome, as expected. Importantly, localization of LANA punctate dots showed similar pattern with both the histones. These data suggest that LANA colocalized with histone H1 as well as H2B. This was further strengthened by performing immunoprecipitation of histones using a myc antibody against the myc tag (to have same antibody affinity) of the GFP fused histones in stable PEL cells to determine relative amounts of co-precipitated LANA. The results showed comparable binding confirming LANA’s ability to bind with both histones, H1 and H2B.

Stable expression of exogenous proteins provides an advantage of getting the expression levels similar to physiological levels as compared to transient transfection where the expressions are comparatively high. Therefore, we have higher confidence in above localization and immunoprecipitation data. Additionally, in our attempt to generate stable cells of 293T with H1-GFP or H2B-GFP and RFP-LANA, we were able to determine the punctate localization of RFP-LANA (although relatively lower number than the control PELs cells) on the chromosomes. Immunoprecipitation of histones with anti-myc antibody co-precipitated LANA with both histones, H1 and H2B after one round of selection. Selection optimizes the expression levels and so confirms that physiological levels are required for fair comparison of binding.

Besides these biochemical binding assays, we attempted to address the binding of LANA with histones in a split GFP complementation assay. This assay has been used for determining in-vivo association of proteins in bacteria, yeast and human cells [46], [68], [69]. LANA1–32 aa with wt CBD fused to GFP-C term and histone H2B fused to GFP-N term showed strong complementation as compared to histone H1 fused to GFP-N terminus confirms their binding observed in other assays. However, a drastic decrease in GFP complementation with a larger domain (1–340 aa) of LANA or full length LANA may suggest that H2B has higher affinity to LANA 1–32 aa in in-vivo conditions. This was also evident by the fact that full length LANA with either wt or mutated CBD (5–15 aa to alanine) showed similar complementation to histones, H1 and H2B suggesting association of both histones with LANA. Not surprising, the association of histone H1 with LANA 1–340 aa as well as full length was consistent thus confirmed its association in-vivo. In another in-vivo protein-protein interaction assay using fluorescence resonance energy transfer (FRET), which relies on the distance between the donor and accepter molecules, we confirmed that the histones H1 and H2B were in close proximity to LANA. These various biochemical assays suggest that binding of LANA with histones may be dependent on the experimental conditions but the in-vivo assays should provide physiologically relevant information on protein-protein interaction.

In-vivo protein-protein interaction assays are powerful tools to determine proteins association/proximity in their native states. Both split GFP-complementation and FRET analysis uses microscopy to precisely analyze the association of proteins by fluorescence of green signals and transfer of energy from the donor to the acceptor fluorophore, respectively. Split GFP complementation assay is a qualitative measure of protein association whereas FRET gives an idea of inter-molecular distance between the associating proteins.

### Proposed Model of LANA Tethering and Transcriptional Regulation

Binding of LANA with histone H1 variants suggested that LANA has the capability of binding to histones. It has been suggested that histone H1 plays a dynamic role in regulation of gene expression [70], and LANA has also been shown to regulate gene expression [41]. Therefore, we hypothesize that binding of LANA to histone H1 not only helps in tethering the viral genome but also is important in regulation of the chromatin structure at the site of its attachment to the host chromatin. LANA has been shown to attach to the chromatins of various genes, recently detected by chromatin immunoprecipitation with anti-LANA antibody and next generation sequencing (ChIP-Seq) [71]. Binding of other nucleosomal proteins with LANA, including MeCP2 and DEK [19], [24], [60], provides precedence for the involvement of other proteins in tethering of the KSHV genome. High mobility group (HMG) proteins are also very abundant protein on chromatin therefore we analyzed their binding with LANA. HMGN proteins do not posses any DNA binding domain therefore they directly bind to the nucleosome core particles through their nucleosome-binding domain [72]–[74]. HMGN proteins are involved in generating as well as maintaining transcriptionally active open chromatins by competing with nucleosome compacting protein, histone H1 [75]. HMGN1 and HMGN2 have also been suggested to be required for the stabilization of nucleosomal core complex of H2A/H2B, H3 and H4 [76], [77]. These proteins bind dynamically to nucleosomes to alter the complexity of the chromatin. Higher order chromatins, which are generated by compaction through histone H1, are inhibitory to chromatin related processes including replication, transcription and DNA repair [78]. Members of the HMG proteins can compete with histone H1 to make the chromatin more accessible for transcription and replication [54], [55]. Since LANA binds to heterochromatin, which has high-density compact chromatin, it may primarily be tethering through histone H1. Additionally, LANA is capable of upregulating transcriptional activity of various cellular and viral genes suggesting it’s binding to open chromatin. Recruitment of HMGN1 through LANA may be important for altering the chromatin architecture to make it more accessible for binding of cellular factors for transcriptional regulation. HMGN1 has also been shown to co-localize with zinc finger protein CCCTC-binding factor (CTCF) binding sites suggesting its role in positioning of nucleosomes around the functional CTCF binding sites in chromatin [59]. Recently, CTCF has been shown to organize the viral genome into chromatin loops to regulate the expression of latent and lytic genes [79]. Since LANA has been shown to regulate the expression of immediate early gene, RTA and latency control region (LANA) [80], we speculate that recruitment of HMGN1 through LANA and their co-localization with CTCF may have implications in controlling latent and lytic gene expression. In conclusion, LANA can bind to various cellular proteins (listed in table 1) in order to achieve diverse functions and the binding is context dependent as it can bind to both open as well as condensed chromatins.

**Table 1:** LANA interacting proteins in episome maintenance.

Proteins	Roles	References
Histone H1	Histones H1 are necessary for the condensation of nucleosome chains into higher order chromatin.	[81]
	LANA was shown to associate with H1 for tethering of viral genome to host chromatins	[2]
RING 3/Brd2	RING3/Brd2 is a member of the Drosophila female sterile gene (fsh)/BET/Brd family of proteins plays rolein nucleosome assembly.	[82],
	Interaction of RING3 with LANA possibly creates a local euchromatic microenvironment around the viralepisomes and anchors the episome to the heterochromatin region.	[29], [30], [26]
MeCP2 and DEK	MeCP2 help regulate gene activity (expression) by modifying chromatin structure. DEK is a DNA-binding phosphoprotein and is involved in changing chromatin topology.	[83], [84], [19], [24]
	LANA associates with MeCP2 and DEK to tether onto the human chromosome.	
HP1/SUV39H1	Heterochromatin protein 1 (HP1) associates with the heterochromatin by binding to methylated lysine9 of histone H3 (H3K9me3). SUV39H1, a human homologue of Drosophila su(var)3–9 was foundto be a histone H3-specific methyltransferase. Methylation by SUV39H1 promotes binding of HP1.	[85],
	SUV39H1 accumulates at TR to heterochromatinize TR region and to allow binding with HP1. LANA-HP1complex at the TR promotes subnuclear localization of the viral genome to the heterochromatin region.	[23], [86], [87]
H2A/H2B	H2A and H2B are the components of the nucleosome core particles and forms octamer with H3 and H4.	[20], [63], [88]
	LANA 5–13 aa interacts with host chromatin by binding to an acidic patch on H2A-H2B within the nucleosome.	
NuMA	Nuclear mitotic apparatus (NuMA) protein interacts with mitotic components including microtubules tosegregate the divided nuclei to the daughter cells.	[89], [90], [21]
	LANA interacts with NuMA and helps in efficient segregation of the viral genome to divided tumor cells.	
Brd4	Brd4 associates with RING3 (a serine/threonine kinase) as well as to the chromosomes during mitosis. Brd4 contains two bromodomains, which have conserved motifs involved in chromatin targeting.	[91], [25]
	Brd4 interacts with LANA and viral episomes in punctate nuclear structures on mitotic chromosomes andmay have role in genome maintenance.	
CENP-F and Bub1	CENP-F, a kinetochore protein is part of the nuclear matrix complex, which links the chromosome tomicrotubule polymers for segregation during mitosis. Bub1 (budding uninhibited by benzimidazole 1)is a serine/threonine kinase, which forms complex with CENP-F and performs multiple tasks duringmitosis to proper inheritance of chromosomes.	[92], [93], [94], [32]
	Interaction of LANA with kinetochore proteins CENP-F and Bub1 is critical for tethering and segregationof KSHV genome into daughter cells.	

## Supporting Information

Figure S1 **Nuclease treatment of the lysates.** **A.** Lysates were supplemented with 100 ng/µl plasmid DNA before treatment with DNase I. Lysates were treated with increasing amounts of DNase I from 5 µg to 50 µg per reaction. A fraction (10%) of the lysate was subjected for the extraction of DNA and resolved on agarose gel to test for proper digestion. 50 µg DNase I was able to completely digest the exogenously added plasmid DNA. **B.** Digestion of cellular DNA was determined by PCR amplification of cellular gene, GAPDH. DNA extracted from untreated lysate showed an amplification of GAPDH but an addition of DNase I showed no amplification confirming degradation of DNA in the lysates. **C.** Lyates were treated with MNase to degrade inter-nucleosomal DNA and to generate mononucleosomes. DNA extracted from untreated (lane 1), 0.5 U MNase treated (lane 3) and 2 U MNase treated (lane 2), were resolved on 2.5% agarose gel. Marker was loaded in lane 4. 0.5 U of MNase was able to partially digest and generate mono-, di- and higher order nucleosomes (lane 3). 2.0 U MNase completely digested the chromatin to yield mononucleosomes and therefore used for subsequent experiments.
(TIF)

Figure S2 **Binding of endogenous histones (H1 and H2B) with LANA 1–32** **aa with wt CBD and its alanine substitution mutants. A.** LANA 1–32 aa and 5–15 alanine mutants cloned in frame with GFP and Myc tag. **B.** GFP- fused LANA1–32 aa wt (lane 1), mutant 5–7 aa (lane 2), mutant 8–10 aa (lane 3), mutant 11–13 aa (lane 4), mutant 14–15 aa (lane 5) and mutant 5–15 aa (lane 6) were transfected into BJAB cells followed by anti-myc immunoprecipitation to IP LANA 1–32 aa and its mutants. Co-precipitating histone H1 and H2B were determined using specific antibodies. Histone H1 and H2B specific bands in input and IP panels are indicated with an arrow. Histone H1 as well as H2B were detected in lane 1, which had wt LANA1–32 aa.
(TIF)

Figure S3 **LANA-FL associated with histone H1 and H2B.** **A.** LANA-myc was co-transfected with histone H1-Flag or H2B-Flag or both histones together into 293T cells. Lysate were divided into two parts, i) untreated **(A)** and ii) DNAse treated **(B),** before immunoprecipitation with anti-myc antibody. Histone H1-Flag and H2B-Flag in the input and IP lanes were detected with anti-Flag WB (Expressed Histones panel). Co-precipitating H1-Flag and H2B-Flag are indicated with arrows. Endogenous histones, H1 and H2B were also detected in the same blot using histone specific antibodies (Endogenous Histones panel) to determine the relative amounts of exogenous verses endogenous histones. Co-precipitating histone H1 in untreated and DNase I treated lysates are indicated with arrows (IB:H1). Histone H2B precipitated from the untreated as well as DNase I treated cell lysates (IB:H2B). **C.** 293T cells were transfected with either myc vector or LANA-myc and immunoprecipitated with anti-myc antibody. The blot probed with anti-HA antibody did not show any signal thus confirmed that H1-HA and H2B-HA signals in previous blots were specific. Detection of endogenous H1 and H2B with specific antibodies showed binding of both H1 and H2B with LANA.
(TIF)

Figure S4 **Co-precipitation of LANA-FL with wt CBD and its alanine mutants with histone H1 and H2B.** **A.** Schematic of LANA showing amino acids of CBD (5–15 aa) and alanine mutants of CBD 5–7 aa (2), 8–10 aa (3), 11–13 aa (4), 14–15 aa (5) and 5–15 aa (6). **B.** Histone H1-Flag was co-transfected with LANA-wt (lane 1) and its mutants in lanes 2–6 (lanes correspond to mutants number in panel A). Histone H1 was immunoprecipitated and detected with anti-Flag antibody (Flag:WB), LC-light chain. Histone H1-Flag was unable to co-precipitate a detectable level of LANA (Myc:WB). Histone H2B-Flag was co-transfected with LANA-wt (lane 1) and its mutants in lanes 2–6 (lanes correspond to mutants number in panel A). Histone H2B was immunoprecipitated and detected with anti-Flag antibody (Flag:WB), LC-light chain and M is marker. Histone H2B-Flag was able to co-precipitate detectable levels of LANA with wt CBD as well all its alanine mutants (Myc:WB). Co-precipitation was specific as the LANA transfected with Flag vector did not precipitate detectable amounts of LANA.
(TIF)

Figure S5 **Histone H1 and H2B fused to GST binds to LANA from KSHV infected PEL cells.** **A.** KSHV negative BJAB and KSHV positive cells, BC3, BCBL1 and JSC1 were lysed in RIPA buffer followed binding with GST (control), GST-H1 and GST-H2B. Complex were washed with 300 mM NaCl to remove loosely bound protein and the remaining protein was resolved on SDS-PAGE for WB. Endogenous LANA binding to GST fusion proteins were detected with anti-LANA WB (IB:LANA), which showed efficient binding with both the histones. Amounts of GST fusion proteins used in this assay are indicated by red triangles (GST panel). **B.** Lysates from the above mentioned cells were treated with 50 ug of DNase I before binding with GST fusion proteins. Bound protein resolved on SDS-PAGE after washing with buffer containing 300 mM of NaCl. Immunoblot with anti-LANA antibody showed its binding with both the histones. Lack of LANA signals in GST lanes shows specificity of the assay.
(TIF)

Figure S6 **Split GFP complementation assay to determine in-vivo association of LANA with histones. A.** Histone H1 or H2B fused with GFP-N term (1–157 aa) were co-transfected with LANA1–32 aa (top panel) and LANA 1–32 aa with alanine substituted 5–15 aa (mut5) of CBD (bottom panel) fused to C-term of GFP (158–234 aa). GFP fluorescence was imaged after 48 h post transfection. LANA 1–32 aa showed strong GFP signals as compared to histone H1. Substitution of CBD residues with alanine in LANA 1–32 aa suppressed its association with both histones H2B as well as H1. **B.** Transfection of GFP-N term (1–157 aa) with GFP-C-term or GFP-C term (158–234 aa) fused with LANA 1–32 aa, LANA 1–32 aa mut5, LANA-N, LANA-FL and LANA-FLmut5 did not show any fluorescence, which confirmed that association of histone with LANA was specific**.** Similarly, GFP-C co-transfected with GFP-N term fused with histone H1 or H2B did not yield any fluorescence. **C.** Western blot with anti-GFP antibody to show expressions of GFP-N term and C-term fused proteins in cells used in imaging. Band of interests are indicated with red arrow.
(TIF)

Figure S7 Cells used for FRET assay, transfected CFP-H1-myc+YFP-LANA-Flag, CFP-H2B-myc+LANA-YFP-Flag and control cells GFP-NLS-myc+LANA-YFP-Flag were selected for 1 month. Lysates from these cells were treated with DNase I before anti-myc immunoprecipitation. Immunoprecipitating GFP-NLS, CFP-H1 and CFP-H2B were detected with anti-myc antibody (IB:myc) and co-precipitating LANA-YFP was detected with both the histones, H1 and H2B but not with control GFP-NLS-myc (IB:Flag).
(TIF)
