# Supplementary figures and images for: Comprehensive Analysis of LANA Interacting Proteins Essential for Viral Genome Tethering and Persistence
Source: PLoS One. 2013 Sep 11;8(9):e74662. doi: 10.1371/journal.pone.0074662 (PMC3770571; doi:10.1371/journal.pone.0074662)

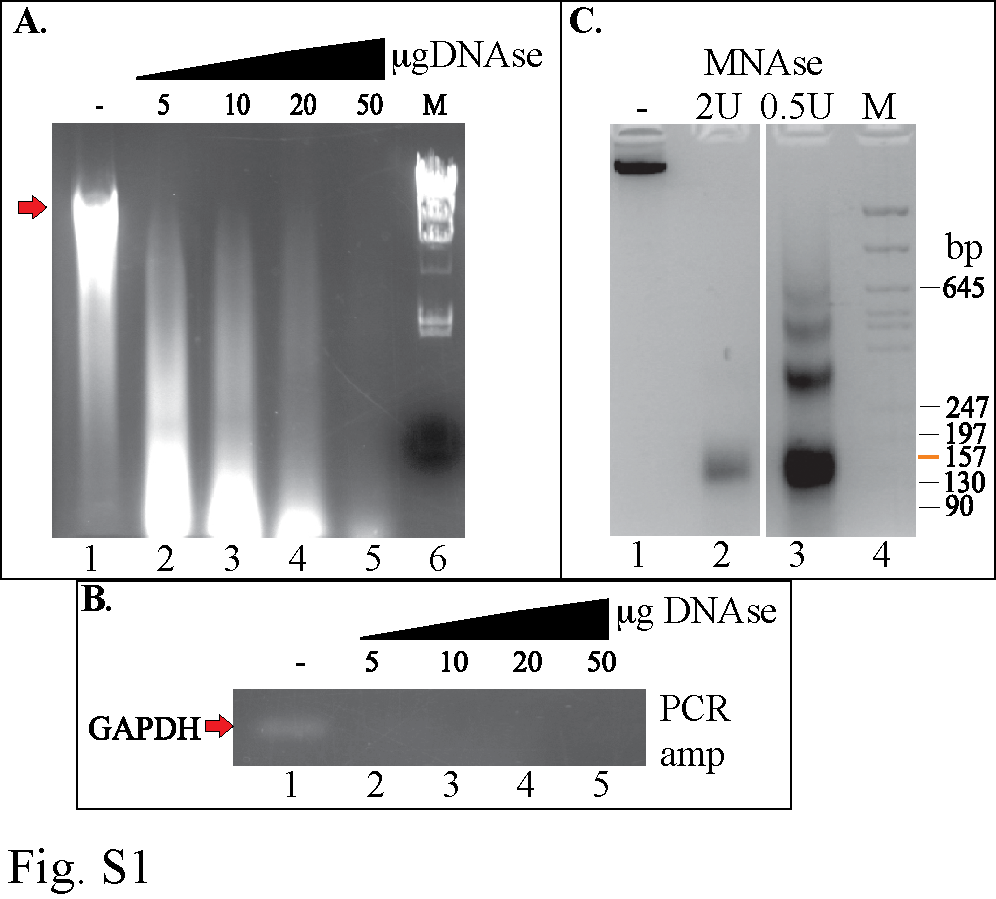

Supplement: Figure S1 — Nuclease treatment of the lysates. A. Lysates were supplemented with 100 ng/µl plasmid DNA before treatment with DNase I. Lysates were treated with increasing amounts of DNase I from 5 µg to 50 µg per reaction. A fraction (10%) of the lysate was subjected for the extraction of DNA and resolved on agarose gel to test for proper digestion. 50 µg DNase I was able to completely digest the exogenously added plasmid DNA. B. Digestion of cellular DNA was determined by PCR amplification of cellular gene, GAPDH. DNA extracted from untreated lysate showed an amplification of GAPDH but an addition of DNase I showed no amplification confirming degradation of DNA in the lysates. C. Lyates were treated with MNase to degrade inter-nucleosomal DNA and to generate mononucleosomes. DNA extracted from untreated (lane 1), 0.5 U MNase treated (lane 3) and 2 U MNase treated (lane 2), were resolved on 2.5% agarose gel. Marker was loaded in lane 4. 0.5 U of MNase was able to partially digest and generate mono-, di- and higher order nucleosomes (lane 3). 2.0 U MNase completely digested the chromatin to yield mononucleosomes and therefore used for subsequent experiments. (TIF) [file pone.0074662.s001.tif]

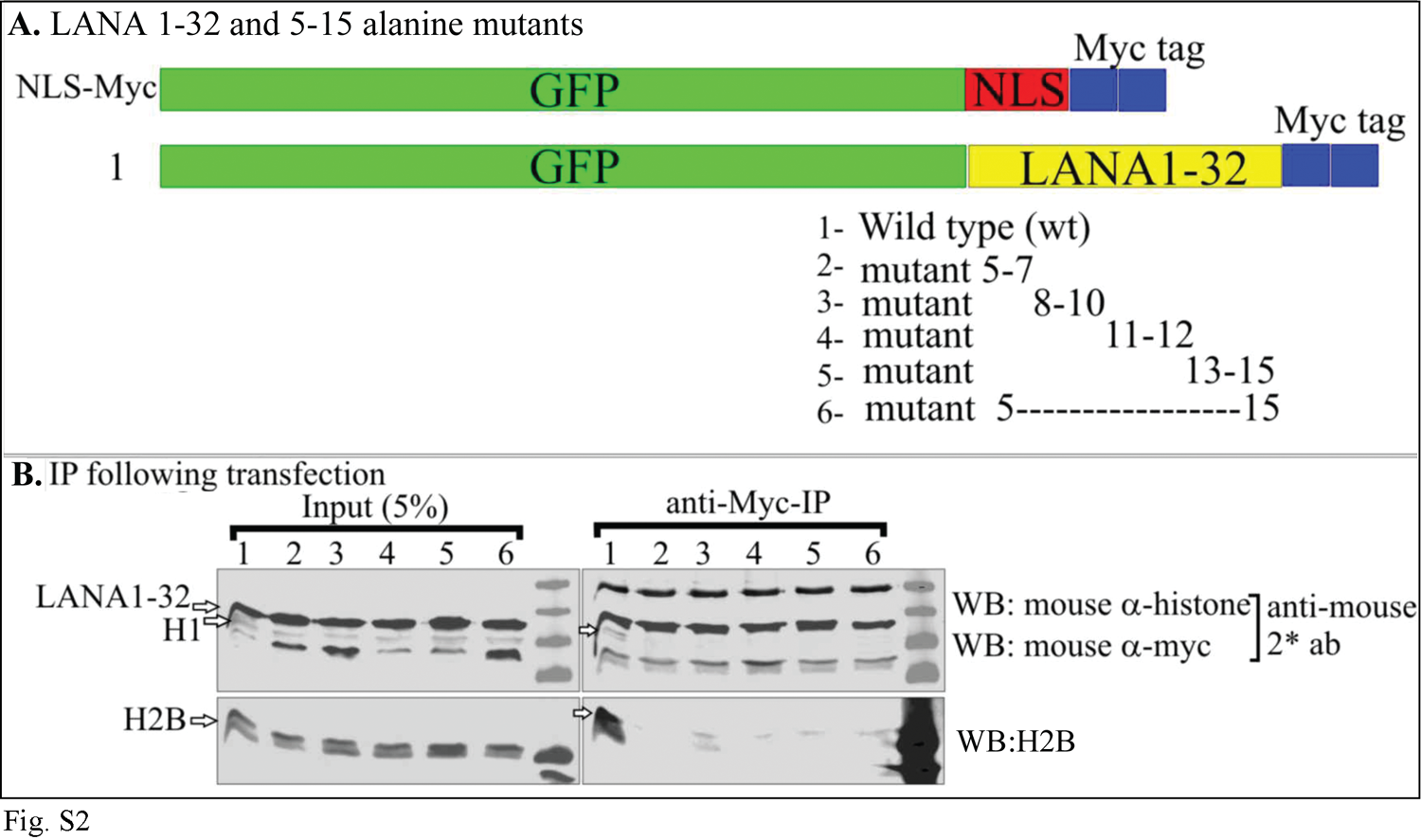

Supplement: Figure S2 — Binding of endogenous histones (H1 and H2B) with LANA 1–32 aa with wt CBD and its alanine substitution mutants. A. LANA 1–32 aa and 5–15 alanine mutants cloned in frame with GFP and Myc tag. B. GFP- fused LANA1–32 aa wt (lane 1), mutant 5–7 aa (lane 2), mutant 8–10 aa (lane 3), mutant 11–13 aa (lane 4), mutant 14–15 aa (lane 5) and mutant 5–15 aa (lane 6) were transfected into BJAB cells followed by anti-myc immunoprecipitation to IP LANA 1–32 aa and its mutants. Co-precipitating histone H1 and H2B were determined using specific antibodies. Histone H1 and H2B specific bands in input and IP panels are indicated with an arrow. Histone H1 as well as H2B were detected in lane 1, which had wt LANA1–32 aa. (TIF) [file pone.0074662.s002.tif]

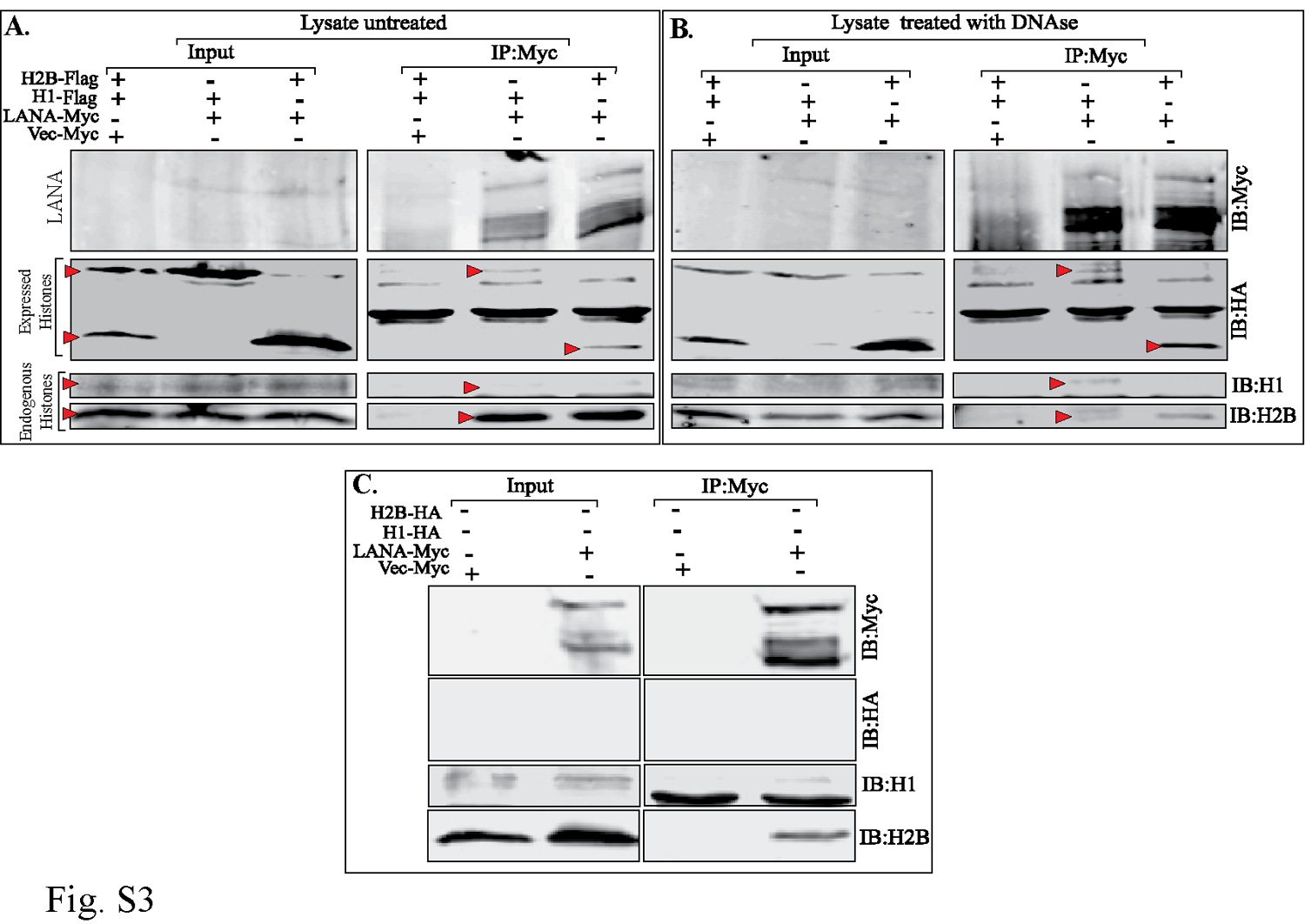

Supplement: Figure S3 — LANA-FL associated with histone H1 and H2B. A. LANA-myc was co-transfected with histone H1-Flag or H2B-Flag or both histones together into 293T cells. Lysate were divided into two parts, i) untreated (A) and ii) DNAse treated (B), before immunoprecipitation with anti-myc antibody. Histone H1-Flag and H2B-Flag in the input and IP lanes were detected with anti-Flag WB (Expressed Histones panel). Co-precipitating H1-Flag and H2B-Flag are indicated with arrows. Endogenous histones, H1 and H2B were also detected in the same blot using histone specific antibodies (Endogenous Histones panel) to determine the relative amounts of exogenous verses endogenous histones. Co-precipitating histone H1 in untreated and DNase I treated lysates are indicated with arrows (IB:H1). Histone H2B precipitated from the untreated as well as DNase I treated cell lysates (IB:H2B). C. 293T cells were transfected with either myc vector or LANA-myc and immunoprecipitated with anti-myc antibody. The blot probed with anti-HA antibody did not show any signal thus confirmed that H1-HA and H2B-HA signals in previous blots were specific. Detection of endogenous H1 and H2B with specific antibodies showed binding of both H1 and H2B with LANA. (TIF) [file pone.0074662.s003.tif]

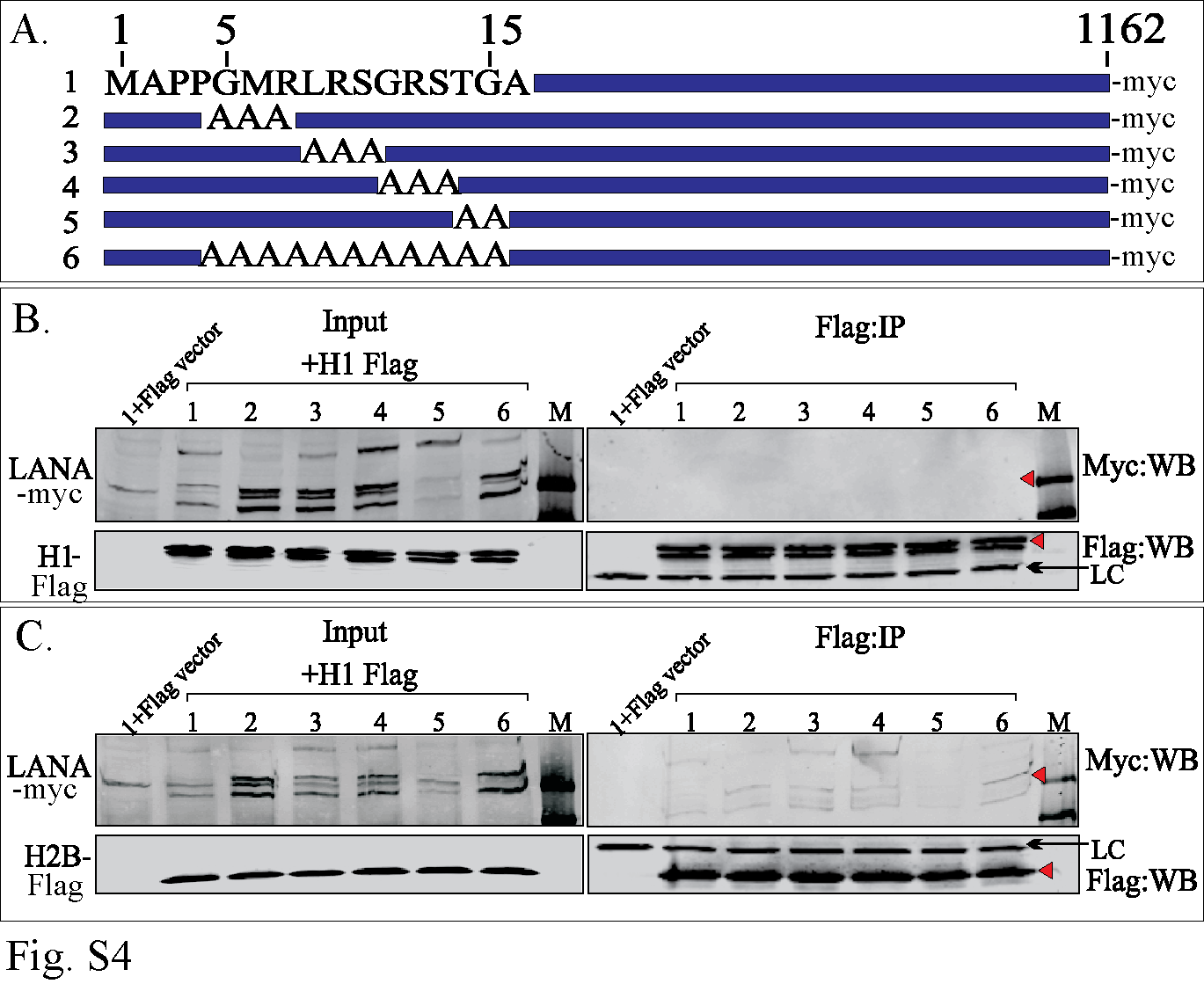

Supplement: Figure S4 — Co-precipitation of LANA-FL with wt CBD and its alanine mutants with histone H1 and H2B. A. Schematic of LANA showing amino acids of CBD (5–15 aa) and alanine mutants of CBD 5–7 aa (2), 8–10 aa (3), 11–13 aa (4), 14–15 aa (5) and 5–15 aa (6). B. Histone H1-Flag was co-transfected with LANA-wt (lane 1) and its mutants in lanes 2–6 (lanes correspond to mutants number in panel A). Histone H1 was immunoprecipitated and detected with anti-Flag antibody (Flag:WB), LC-light chain. Histone H1-Flag was unable to co-precipitate a detectable level of LANA (Myc:WB). Histone H2B-Flag was co-transfected with LANA-wt (lane 1) and its mutants in lanes 2–6 (lanes correspond to mutants number in panel A). Histone H2B was immunoprecipitated and detected with anti-Flag antibody (Flag:WB), LC-light chain and M is marker. Histone H2B-Flag was able to co-precipitate detectable levels of LANA with wt CBD as well all its alanine mutants (Myc:WB). Co-precipitation was specific as the LANA transfected with Flag vector did not precipitate detectable amounts of LANA. (TIF) [file pone.0074662.s004.tif]

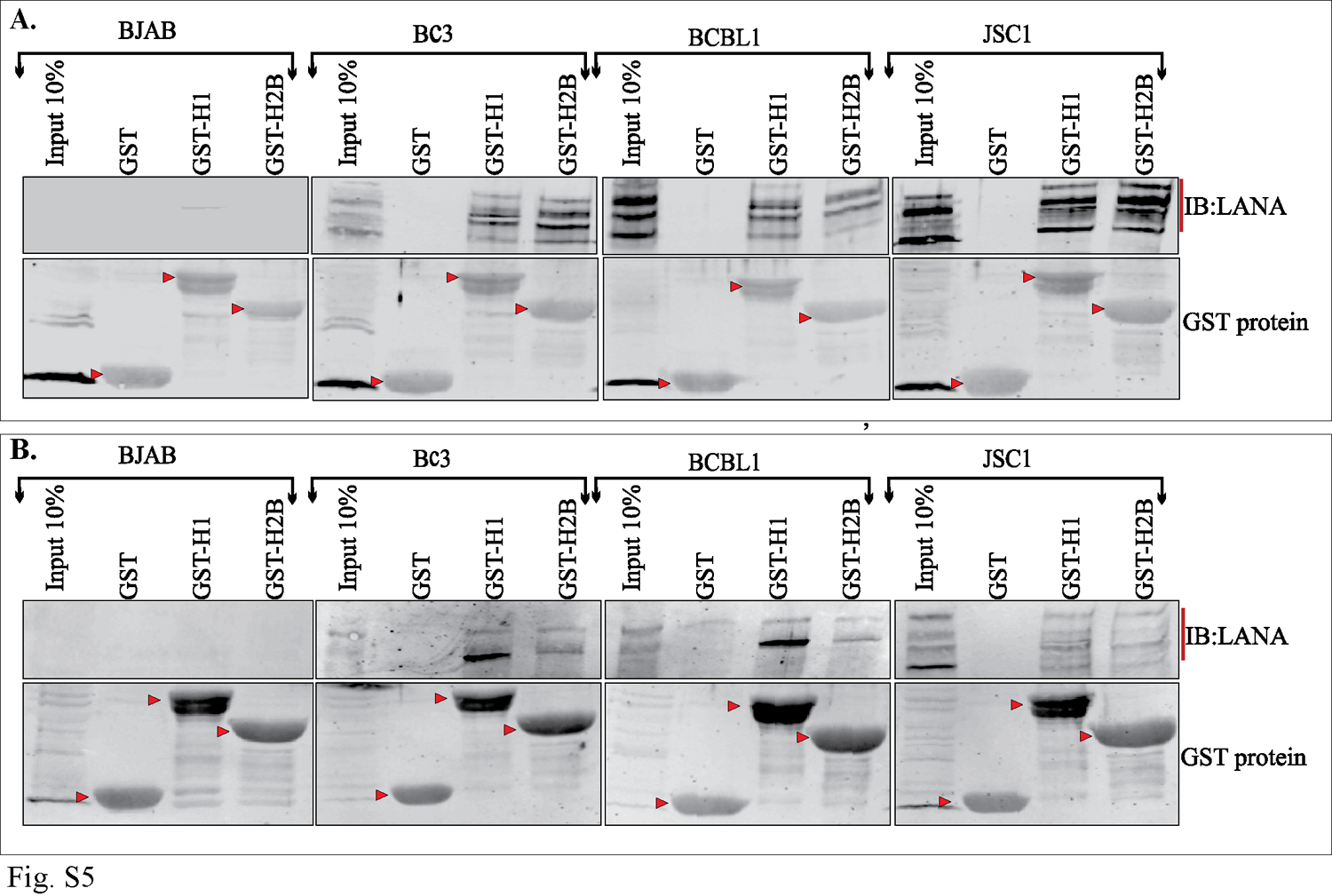

Supplement: Figure S5 — Histone H1 and H2B fused to GST binds to LANA from KSHV infected PEL cells. A. KSHV negative BJAB and KSHV positive cells, BC3, BCBL1 and JSC1 were lysed in RIPA buffer followed binding with GST (control), GST-H1 and GST-H2B. Complex were washed with 300 mM NaCl to remove loosely bound protein and the remaining protein was resolved on SDS-PAGE for WB. Endogenous LANA binding to GST fusion proteins were detected with anti-LANA WB (IB:LANA), which showed efficient binding with both the histones. Amounts of GST fusion proteins used in this assay are indicated by red triangles (GST panel). B. Lysates from the above mentioned cells were treated with 50 ug of DNase I before binding with GST fusion proteins. Bound protein resolved on SDS-PAGE after washing with buffer containing 300 mM of NaCl. Immunoblot with anti-LANA antibody showed its binding with both the histones. Lack of LANA signals in GST lanes shows specificity of the assay. (TIF) [file pone.0074662.s005.tif]

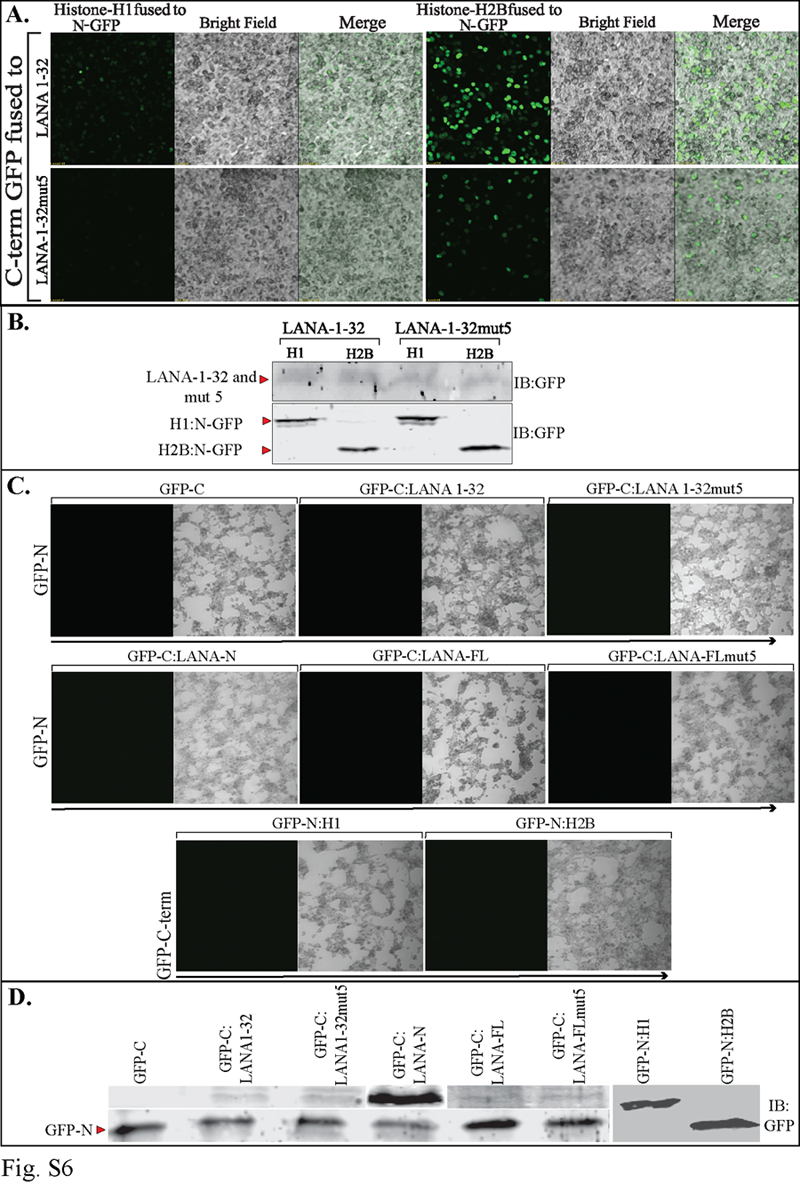

Supplement: Figure S6 — Split GFP complementation assay to determine in-vivo association of LANA with histones. A. Histone H1 or H2B fused with GFP-N term (1–157 aa) were co-transfected with LANA1–32 aa (top panel) and LANA 1–32 aa with alanine substituted 5–15 aa (mut5) of CBD (bottom panel) fused to C-term of GFP (158–234 aa). GFP fluorescence was imaged after 48 h post transfection. LANA 1–32 aa showed strong GFP signals as compared to histone H1. Substitution of CBD residues with alanine in LANA 1–32 aa suppressed its association with both histones H2B as well as H1. B. Transfection of GFP-N term (1–157 aa) with GFP-C-term or GFP-C term (158–234 aa) fused with LANA 1–32 aa, LANA 1–32 aa mut5, LANA-N, LANA-FL and LANA-FLmut5 did not show any fluorescence, which confirmed that association of histone with LANA was specific. Similarly, GFP-C co-transfected with GFP-N term fused with histone H1 or H2B did not yield any fluorescence. C. Western blot with anti-GFP antibody to show expressions of GFP-N term and C-term fused proteins in cells used in imaging. Band of interests are indicated with red arrow. (TIF) [file pone.0074662.s006.tif]

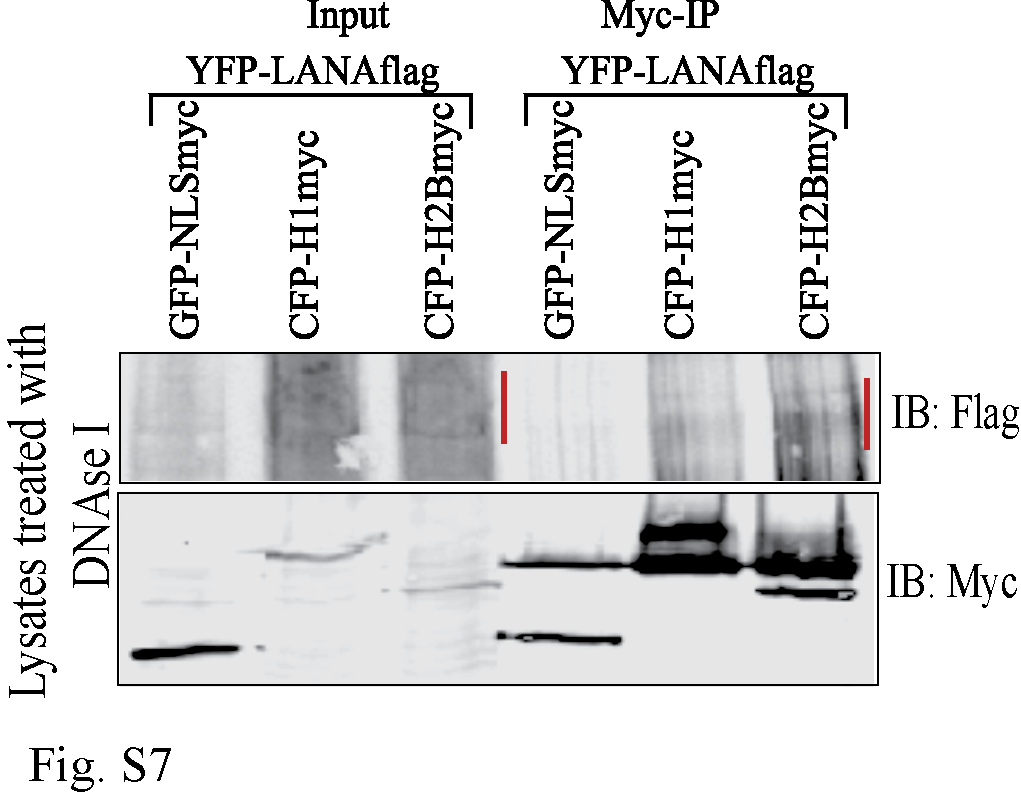

Supplement: Figure S7 — Cells used for FRET assay, transfected CFP-H1-myc+YFP-LANA-Flag, CFP-H2B-myc+LANA-YFP-Flag and control cells GFP-NLS-myc+LANA-YFP-Flag were selected for 1 month. Lysates from these cells were treated with DNase I before anti-myc immunoprecipitation. Immunoprecipitating GFP-NLS, CFP-H1 and CFP-H2B were detected with anti-myc antibody (IB:myc) and co-precipitating LANA-YFP was detected with both the histones, H1 and H2B but not with control GFP-NLS-myc (IB:Flag). (TIF) [file pone.0074662.s007.tif]
